# Efficacy of 10-valent pneumococcal non-typeable *Haemophilus influenzae* protein D conjugate vaccine against acute otitis media and nasopharyngeal carriage in Panamanian children – A randomized controlled trial

**DOI:** 10.1080/21645515.2017.1287640

**Published:** 2017-02-25

**Authors:** Xavier Sáez-Llorens, Stella Rowley, Digna Wong, Mirna Rodríguez, Arlene Calvo, Marisol Troitiño, Albino Salas, Vielka Vega, Maria Mercedes Castrejón, Patricia Lommel, Thierry G. Pascal, William P. Hausdorff, Dorota Borys, Javier Ruiz-Guiñazú, Eduardo Ortega-Barría, Juan Pablo Yarzabal, Lode Schuerman

**Affiliations:** a Department of Infectious Diseases, Hospital del Niño, Panama City, Panama; Distinguished Member of the SNI, Senacyt, Panama; b Department of Otorhinolaryngology Hospital del Niño, Panama City, Panama; c Instituto de Investigaciones Científicas y Servicios de Alta Tecnología (INDICASAT), Panama City, Panama; d Health Research International, Panama City, Panama; e GSK Vaccines, Panama City, Panama; f GSK Vaccines, Wavre, Belgium

**Keywords:** acute otitis media, children, efficacy, nasopharyngeal carriage, pneumococcal conjugate vaccination

## Abstract

We previously reported 10-valent pneumococcal non-typeable *Haemophilus influenzae* (NTHi) protein D conjugate vaccine (PHiD-CV) efficacy in a double-blind randomized trial (ClinicalTrials.gov: NCT00466947) against various diseases, including acute otitis media (AOM). Here, we provide further analyses. In the Panamanian subset, 7,359 children were randomized (1:1) to receive PHiD-CV or control vaccine at age 2/4/6 and 15–18 months. Of these, 2,000 had nasopharyngeal swabs collected. AOM cases were captured when parents sought medical attention for children with AOM symptoms; surveillance was enhanced approximately 2 y into the study through regular telephone calls or home visits by study personnel, who advised parents to visit the clinic if their child had AOM symptoms. Mean follow-up was 31.4 months. Clinical AOM (C-AOM) cases were assessed by physicians and confirmed by otorhinolaryngologists. Middle ear fluid samples, taken from children with C-AOM after specific informed consent, and nasopharyngeal samples were cultured for pathogen identification. For 7,359 children, 2,574 suspected AOM cases were assessed by a primary healthcare physician; 649 cases were C-AOM cases as per protocol definition. From the 503 MEF samples collected, 158 resulted in a positive culture. In the intent-to-treat cohort (7,214 children), PHiD-CV showed VE against first C-AOM (24.0% [95% CI: 8.7, 36.7]) and bacterial (B-AOM) episodes (48.0% [20.3, 66.1]) in children <24 months, which declined thereafter with age. Pre-booster VE against C-AOM was 30.7% [12.9, 44.9]; post-booster, −6.7% [−36.4, 16.6]. PHiD-CV VE was 17.7% [−6.1, 36.2] against moderate and 32.7% [−20.5, 62.4] against severe C-AOM. VE against vaccine-serotype pneumococcal NPC was 31.2% [5.3, 50.3] 3 months post-booster, and 25.6% [12.7, 36.7] across all visits. NTHi colonization rates were low and no significant reduction was observed. PHiD-CV showed efficacy against C-AOM and B-AOM in children younger than 24 months, and reduced vaccine-serotype NPC.

## Introduction

Acute otitis media (AOM) is one of the most common bacterial diseases in young children, with a peak of incidence at the age of 6–18 months.^1^ In many countries, AOM remains the main reason for antimicrobial prescriptions in children, contributing to the development of antibiotic resistance, and resulting in a major burden on healthcare systems.^2-4^ In Latin America, limited data on AOM incidence have shown a range of 0.01–0.36 episodes per person-year in children younger than 5 y.^5^ In Panama, we have reported a clinically-confirmed AOM (C-AOM) incidence rate of 0.036 episodes per child-year in the control group of the Clinical Otitis Media and Pneumonia Study (COMPAS).^6^ Previous studies of middle ear fluid (MEF) samples have shown that *Streptococcus pneumoniae* and non-typeable *Haemophilus influenzae* (NTHi) are the main bacterial pathogens responsible for pediatric AOM in Argentina, Colombia, Mexico, Venezuela, Costa Rica and Chile, in line with observations from other regions worldwide.^7-12^

Until recently, efficacy/effectiveness against AOM was evaluated in double-blind randomized studies for only one of the 3 licensed pneumococcal conjugate vaccines (PCVs), the 7-valent CRM_197_-conjugate vaccine (7vCRM; *Prevenar/Prevnar™*, Pfizer Inc., NY)..^13,14^ For the 10-valent pneumococcal NTHi protein D conjugate vaccine (PHiD-CV; *Synflorix™*, GSK Vaccines, Rixensart, Belgium), a recently published randomized controlled trial (RCT) in Finland reported a trend towards reduced AOM.^15^ For the 13-valent CRM_197_-conjugate vaccine (PCV13; *Prevenar 13/Prevnar 13™*, Pfizer Inc., NY), no RCTs assessing AOM have yet been reported, although impact on AOM has been assessed in post-marketing studies.^16-18^ However, as comparisons between RCTs and post-marketing studies are difficult, and especially so for AOM due to the presence of various confounding factors (e.g. methodological aspects, local epidemiology, geographical factors), we have focused on RCTs to provide context for our trial. While 7vCRM has now largely been replaced by PCV13 and PHiD-CV, 7vCRM was extensively assessed in RCTs, thus allowing to compare those results with our current findings. Additionally, these 7vCRM studies report PCV impact in regions without prior PCV use; similarly, our setting in Panama knew no prior PCV use.

In 7vCRM studies, the overall AOM incidence was reduced by 6–7% in children vaccinated with 7vCRM, although more important reductions were reported for more severe AOM cases and for children with recurrent AOM episodes.^13,19^ Analyses of tympanocentesis samples showed that the observed reduction was due to a significant impact on vaccine-type pneumococcal AOM, partly mitigated by an increase in non-vaccine-type pneumococcal AOM and with a trend towards increased NTHi AOM.^14,20^ A longer follow-up of one of these studies found that efficacy against all-cause AOM seemed short-lived for that study, with evidence of significant waning beginning after the second year.^21^ Additionally, a third 7vCRM study could not detect any efficacy against all-cause clinically-diagnosed otitis media, although an efficacy of 64% against vaccine-serotype pneumococcal otitis media was observed.^22^ Subsequent AOM effectiveness analyses following widespread introduction of 7vCRM reported a wide range of results varying from −7% to 48%, likely confounded by a number of non-vaccine factors.^23,24^

Previously, we reported efficacy against AOM in the COMPAS trial for PHiD-CV, together with efficacy against other disease endpoints. Our double-blind randomized clinical trial, conducted in Latin America, showed PHiD-CV efficacy of 19.0% (95% confidence interval [CI]: 4.4, 31.4) against first episodes of C-AOM, and 69.9% (95% CI: 29.8, 87.1) against C-AOM caused by vaccine pneumococcal serotypes in the intent-to-treat analysis.^6^ However, important questions regarding the efficacy of PHiD-CV against AOM, including the level of protection in different age groups and efficacy against AOM with various degrees of severity remained unanswered. These questions are addressed in the current manuscript, as descriptive results indicating trends based on exploratory analyses. For the majority of subanalyses, the results are presented for the intent-to-treat cohort as it included more cases than the per-protocol cohort.

Furthermore, the impact of PHiD-CV on nasopharyngeal carriage is relevant, since vaccines directed against the major bacterial pathogens causing AOM could prevent the disease by at least 2 mechanisms: a direct one due to antibodies exuded into the MEF, as well as indirectly via impact on nasopharyngeal colonization of those pathogens. Reductions in nasopharyngeal carriage of vaccine pneumococcal serotypes were seen with 7vCRM, with a significant decrease in vaccine-serotype carriage after the 7vCRM booster (from 18% [95% CI: 13–23] at 12–15 months of age to 9% [95% CI: 5–13] at 15–18 months of age, p = 0.001) in a study in the US,^25^ a lower likelihood for 7vCRM vaccinees to be colonized with vaccine serotypes (odds ratio 0.40 [95% CI: 0.38–0.99]) in a study of American Indian infants,^26^ and significant reductions (p < 0.001) in vaccine-type pneumococcal carriage in the second year of life with both a 2-dose and a 2+1-dose 7vCRM schedule.^27^ Varying levels of protection against vaccine-serotype carriage have been observed with different numbers of 7vCRM doses.^28-30^ The observed impact on vaccine-type carriage was accompanied by an increase in non-vaccine-types; no impact on NTHi carriage has been reported.^25-27,30^ However, the quantitative and temporal relationship between carriage impact and AOM impact appears complex.^31,32^ Here, we report for the first time a parallel assessment of the impact of PHiD-CV on both bacteriologically-confirmed AOM (B-AOM) and nasopharyngeal carriage of *S. pneumoniae, H. influenzae* and other bacterial pathogens, evaluated in a subset of children from the COMPAS trial.

## Results

### Study population

**Figure 1.**
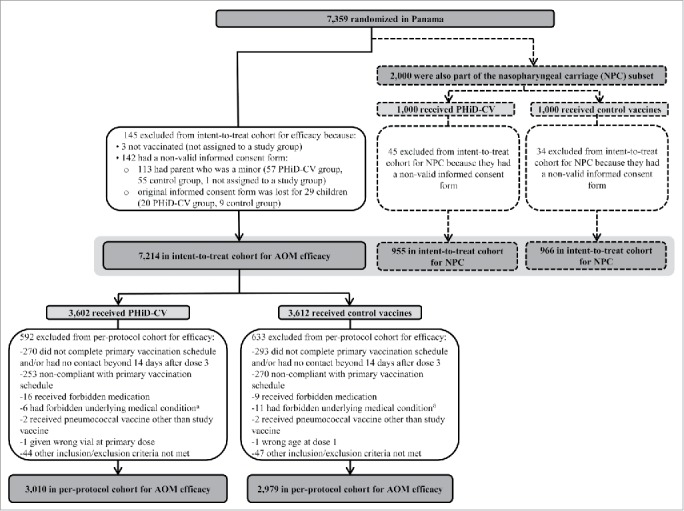
Trial profile for children included in the analysis of acute otitis media (AOM) and nasopharyngeal carriage (NPC). Footnote: Elimination criteria shown for one reason only although more than one reason for elimination could apply per child. For children part of the carriage subset, both efficacy against AOM and impact on nasopharyngeal carriage were assessed. Note that overall, 142 children were excluded from the intent-to-treat cohort due to non-valid informed consent forms, among them; the 79 children excluded also from the NPC intent-to-treat cohort. ^a^ Forbidden underlying medical conditions included, but were not limited to: major congenital defects or serious chronic illness, and confirmed or suspected immunosuppressive or immunodeficient condition. AOM, acute otitis media; NPC, nasopharyngeal carriage.

The numbers of children included in the intent-to-treat and per-protocol cohorts are shown in Fig. 1. Demographic characteristics were comparable in the PHiD-CV and Control groups, as well as in the intent-to-treat and per-protocol cohorts (Table 1). The vast majority (6143 of 7214 [85%] children in the intent-to-treat cohort and 5568 of 5989 [93%] in the per-protocol cohort) received at least one dose of influenza vaccine (seasonal and/or pandemic).

**Table 1.** Demographic characteristics of the trial participants included in the analysis of AOM and nasopharyngeal carriage.

		Intent-to-treat cohort for AOM		Per-protocol cohort for AOM		Intent-to-treat cohort for NPC	
Characteristic	Category	PHiD-CV (N = 3,602)	Control (N = 3,612)	PHiD-CV (N = 3,010)	Control (N = 2,979)	PHiD-CV (N = 955)	Control (N = 966)
Mean age ± SD	At dose 1 (weeks)	9.0 ± 1.3	9.0 ± 1.3	9.0 ± 1.3	9.0 ± 1.3	8.9 ± 1.3	8.9 ± 1.3
	At booster dose (months)	15.8 ± 1.8	15.8 ± 2.0	15.7 ± 1.7	15.7 ± 1.8	16.0 ± 2.2	16.1 ± 2.5
Sex	Female, n (%)	1,762 (48.9)	1,775 (49.1)	1,478 (49.1)	1,464 (49.1)	460 (48.2)	482 (49.9)
	Male, n (%)	1,840 (51.1)	1,837 (50.9)	1,532 (50.9)	1,515 (50.9)	495 (51.8)	484 (50.1)

N, number of children; SD, standard deviation; n (%), number of children in a given category; AOM, acute otitis media; NPC, nasopharyngeal carriage.

#### AOM efficacy cohorts

As previously presented, 7,359 children were randomized to receive PHiD-CV or control; 7,214 children were included in the intent-to-treat cohort for AOM efficacy and 5,989 in the per-protocol cohort for AOM efficacy (Fig. 1).^6^

Mean efficacy follow-up for AOM was 28.2 months (range, 0.0–40.0 months) for the per-protocol cohort and 31.4 months (range, 0.0–45.0 months) for the intent-to-treat cohort.

#### Nasopharyngeal carriage cohorts

**Figure 2.**
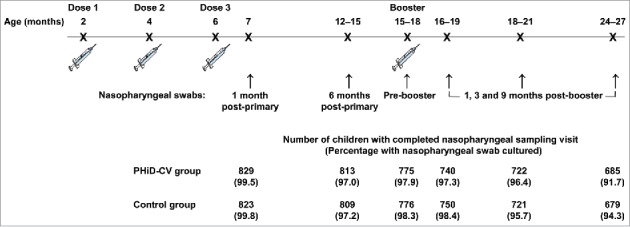
Timing and compliance for nasopharyngeal swab collection (total vaccinated cohort for carriage analysis).

Of the 2,000 children (1,000 in the PHiD-CV group and 1,000 in the Control group) enrolled in this subset of the AOM efficacy cohort, 1,921 children (955 in the PHiD-CV group and 966 in the Control group) were included in the intent-to-treat cohort for analysis of carriage (Fig. 1). The number of children from whom nasopharyngeal swabs were taken ranged from 1,652 children (829 in the PHiD-CV group and 823 in the Control group) at one month post-primary vaccination to 1,364 children (685 in the PHiD-CV group and 679 in the Control group) at 9 months post-booster vaccination. Compliance for collection of nasopharyngeal swabs and percentages of children with nasopharyngeal swabs cultured were comparable between groups at each time point (Fig. 2).

### Occurrence of AOM

In total, 2,574 AOM cases were assessed by a primary healthcare physician (PHCP); of these, 2,215 cases (86%) were seen by an ear, nose and throat (ENT) specialist. Out of the 1,813 AOM cases assessed by PHCP with at least 2 reported symptoms, 649 cases (36%) after ENT assessment were considered as C-AOM cases as per protocol definition (at least one otoscopic sign reported by ENT and at least 2 clinical symptoms reported by PHCP). In addition, of a total of 503 MEF collected, 345 samples (69%) turned out to be documented as culture-negative cases.

From an analytical point of view, a new AOM episode was defined when ≥ 30 d elapsed from the start of a previous episode. During the intent-to-treat follow-up, 637 C-AOM episodes (294 in the PHiD-CV group and 343 in the Control group), occurring in 562 children (8% of the enrolled children), were confirmed (Table 2). The incidence of C-AOM in the Control group corresponded to 0.036 episodes per child-year.

A total of 117 culture-positive B-AOM episodes were reported in the intent-to-treat cohort; 56 with *S. pneumoniae*, 45 with *H. influenzae*, 9 with *S. aureus*, 8 with *Group A streptococcus*, 2 with *M. catarrhalis* and 1 with other bacteria (*Enterobacter cloacae*), with 4 of the 117 episodes having both *S. pneumoniae* and *H. influenzae* bacteria; 112 of these B-AOM episodes were first episodes (Table 2).

**Table 2.** Occurrence of AOM episodes in the intent-to-treat and per-protocol analyses.

	First AOM episodes, number (%)				All AOM episodes,^a^ number (%)			
	Intent-to-treat analysis		Per-protocol analysis		Intent-to-treat analysis		Per-protocol analysis	
Case definition	PHiD-CV group (N = 3,602)	Control group (N = 3,612)	PHiD-CV group (N = 3,010)	Control group (N = 2,979)	PHiD-CV group (N = 3,602)	Control group (N = 3,612)	PHiD-CV group (N = 3,010)	Control group (N = 2,979)
C-AOM	254 (7.1)	308 (8.5)	204 (6.8)	239 (8.0)	294 (8.2)	343 (9.5)	226 (7.5)	257 (8.6)
B-AOM	45 (1.3)	67 (1.9)	32 (1.1)	45 (1.5)	47 (1.3)	70 (1.9)	32 (1.1)	46 (1.5)
Pneumococcal C-AOM	17 (0.5)	38 (1.1)	12 (0.4)	27 (0.9)	18 (0.5)	38 (1.1)	12 (0.4)	27 (0.9)
Vaccine serotype C-AOM	7 (0.2)	23 (0.6)	6 (0.2)	18 (0.6)	7 (0.2)	23 (0.6)	6 (0.2)	18 (0.6)
Vaccine-related serotypes^b^	5 (0.1)	7 (0.2)	3 (0.1)	4 (0.1)	5 (0.1)	7 (0.2)	3 (0.1)	4 (0.1)
Other serotype C-AOM	6 (0.2)	7 (0.2)	3 (0.1)	4 (0.1)	6 (0.2)	7 (0.2)	3 (0.1)	4 (0.1)
*H. influenzae* C-AOM	20 (0.6)	24 (0.7)	12 (0.4)	14 (0.5)	20 (0.6)	25 (0.7)	12 (0.4)	14 (0.5)
NTHi C-AOM	19 (0.5)	24 (0.7)	12 (0.4)	14 (0.5)	19 (0.5)	25 (0.7)	12 (0.4)	14 (0.5)
*Staphylococcus aureus*^c^	3 (0.1)	6 (0.2)	2 (0.1)	3 (0.1)	3 (0.1)	6 (0.2)	2 (0.1)	3 (0.1)
*Group A streptococcus*	4 (0.1)	4 (0.1)	4 (0.1)	4 (0.1)	4 (0.1)	4 (0.1)	4 (0.1)	4 (0.1)
*Moraxella catarrhalis*	2 (0.1)	0 (0)	1 (0.0)	0 (0)	2 (0.1)	0 (0)	1 (0.0)	0 (0)

a All AOM episodes, including multiple episodes in the same child.

b Pneumococcal serotype 6A, 18B, 19A or 23A.

c Among 9 detected *S. aureus,* 1 was mixed with *Pseudomonas aeruginosa* (thus, *S. aureus* should be considered as contaminant), while the remaining 8 with *S. aureus* as only pathogen were 50% from middle-ear fluid and 50% from spontaneous drainage.

%, percentage of episodes in the specified category; N, number of children; AOM, acute otitis media; C-AOM, clinically-confirmed AOM; B-AOM, bacteriologically-confirmed AOM; NTHi, non-typeable *Haemophilus influenzae*; intent-to-treat analysis, follow-up starting at the time of first vaccination; per-protocol analysis, follow-up starting 2 weeks post-dose 3.

AOM severity was categorized according to the 5 symptoms used in the clinical/otologic scale developed by Dagan *et al.*^33^ Among all C-AOM episodes for which the presence or absence of all 5 symptoms on the Dagan scale were recorded (approximately 87% of all C-AOM episodes), 46% were categorized as mild, 45% were moderate, and few episodes (9%) were severe (Table 3). The majority of C-AOM and B-AOM episodes occurred in children aged less than 24 months. Moreover, most were first AOM episodes; thus, efficacy results for all AOM episodes were similar to those for first episodes and are presented in Tables 3 and 4.

**Table 3.** Subanalyses of vaccine efficacy (VE) against C-AOM: by age, pre-/post-booster vaccination, episode severity and by sex (intent-to-treat analysis).

	First C-AOM episodes								All C-AOM episodes^a^					
	Number of children		Follow-up time, person-years^b^		Incidence, per 100,000 person-years			Number of episodes		Follow-up time, person-years^c^		Incidence, per 100,000 person-years		
Group	PHiD-CV	Control	PHiD-CV	Control	PHiD-CV	Control	VE against first episodes, % (95% CI)	PHiD-CV	Control	PHiD-CV	Control	PHiD-CV	Control	VE against all episodes, % (95% CI)
**Total**	**254**	**308**	**9018.3**	**8835.1**	**2816.5**	**3486.1**	**19.0 (4.4, 31.4)**	**294**	**343**	**9475.3**	**9412.8**	**3102.8**	**3644.0**	14.8 (−1.0, 28.2)
**AOM by sex**^d^														
Females	119	140	4370.0	4329.6	2723.1	3233.6	15.9 (−7.4; 34.1)	139	150	4589.7	4590.5	3028.5	3267.6	7.57 (−18.40, 27.85)
Male	135	168	4648.3	4505.5	2904.3	3728.8	21.7 (1.8; 37.6)	155	193	4885.6	4822.3	3172.6	4002.2	20.54 (−0.32, 37.07)
**Age**														
2–11 months	104	141	2786.8	2762.3	3731.9	5104.4	26.9 (5.9, 43.3)	115	150	2824.7	2812.6	4071.2	5333.1	23.7 (1.3, 41.0)
12–23 months	99	122	3023.8	2956.5	3274.0	4126.5	19.7 (−4.7, 38.4)	114	135	3176.7	3155.2	3588.6	4278.7	16.1 (−8.1, 34.8)
<24 months	203	263	5810.6	8873.8	3493.6	2963.8	24.0 (8.7, 36.7)	229	285	6001.4	5967.8	3815.8	4775.6	20.1 (3.8, 33.6)
24–35 months	47	42	2474.3	2390.8	1899.6	1756.7	−10.5 (−67.5, 27.1)	60	54	2670.3	2636.2	2246.9	2048.4	−9.7 (−60.4, 25.0)
≥ 36 months	4	3	733.4	725.4	545.4	545.4	−33.8 (−497.7, 70.1)	5	4	803.6	808.8	622.2	494.6	−25.3 (−366.5, 66.4)
**Pre-/post-booster**														
Pre-booster	124	177	3967.5	3925.8	3125.4	4508.6	30.7 (12.9, 44.9)	140	191	4050.2	4039.4	3456.6	4728.4	27.0 (7.8, 42.2)
Post-booster	132	123	5425.1	5373.4	2433.1	2289.1	−6.7 (−36.4, 16.6)	140	128	5425.1	5373.4	2580.6	2382.1	−8.5 (−39.0, 15.4)
**AOM severity: complete reporting of all symptoms^e^**														
Mild	119	120	9278.7	9202.6	1282.5	1304.0	1.7 (−26.7, 23.7)	127	127	9475.3	9412.8	1340.3	1349.2	0.7 (−28.5, 23.2)
Moderate	109	131	9269.9	9156.6	1175.8	1430.7	17.7 (−6.1, 36.2)	114	137	9475.3	9412.8	1203.1	1455.5	17.3 (−6.9, 36.0)
Severe	19	28	9439.5	9359.0	201.3	299.2	32.7 (−20.5, 62.4)	19	28	9475.3	9412.8	200.5	297.5	32.6 (−20.4, 62.3)
**AOM severity: including incomplete reporting of symptoms (≥ 1 symptom)**^f^														
Mild	158	178	9198.1	9091.3	1717.8	1957.9	12.2 (−8.8, 29.1)	175	191	9475.3	9412.8	1846.9	2029.2	9.0 (−13.2, 26.8)
Moderate	126	146	9237.0	9135.0	1364.1	1598.3	14.7 (−8.3, 32.8)	133	154	9475.3	9412.8	1403.6	1636.1	14.2 (−9.2, 32.5)
Severe	22	28	9434.9	9359.0	233.2	299.2	22.1 (−36.1, 55.4)	22	28	9475.3	9412.8	232.2	297.5	22.0 (−36.0, 55.3)

AOM, acute otitis media; C-AOM, clinically-confirmed AOM; CI, confidence interval; post-booster, at the time of or after booster vaccination; intent-to-treat analysis, follow-up starting at the time of first vaccination. Vaccine efficacy was calculated as (1 – hazard ratio) x 100 using a Cox (first episodes) or generalized Cox (all episodes) regression model.

a All C-AOM episodes, including multiple episodes in the same child.

b Follow-up time for first episodes calculated as sum of follow-up periods of each child, censored at the first occurrence of a respective end point event.

c Follow-up time for all episodes calculated as sum of follow-up periods of each child, from dose 1 up to end of follow-up.

d Post-hoc analysis

e C-AOM episodes with complete reporting of information for all 5 symptoms (∼80% of all reported C-AOM cases) on the clinical/otologic scale developed by Dagan *et al.^33^*

f C-AOM episodes with information for at least 1 symptom on the clinical/otologic scale developed by Dagan *et al.^33^*; unrecorded symptoms were assumed not to be present in the patient.

**Table 4.** Vaccine efficacy (VE) against B-AOM by age and pre-/post-booster vaccination (intent-to-treat cohort).

	First B-AOM episodes							All B-AOM episodes^a^						
	Number of children with ≥ 1 episode		Follow-up time, person-years		Incidence, per 100,000 person-years			Number of episodes		Follow-up time, person-years		Incidence, per 100,000 person-years		
Group	PHiD-CV	Control	PHiD-CV	Control	PHiD-CV	Control	VE against first episodes, % (95% CI)	PHiD-CV	Con-trol	PHiD-CV	Control	PHiD-CV	Control	VE against all episodes, % (95% CI)
**Total**	**45**	**67**	**9396.9**	**9271.0**	**478.9**	**722.7**	**33.6 (3.2, 54.5)**	**47**	**70**	**9475.3**	**9412.8**	**496.0**	**743.7**	33.3 (2.26, 54.5)
**Age**														
2–11 months	20	35	2817.7	2798.3	709.8	1250.8	43.3 (1.7, 67.3)	21	35	2824.7	2812.6	743.4	1244.4	40.3 (−4.0, 65.7)
12–23 months	12	26	3150.1	3105.2	380.9	837.3	54.2 (9.3, 76.9)	13	27	3176.7	3155.2	409.2	855.7	52.1 (6.7, 75.5)
<24 months	32	61	5967.8	5903.5	536.2	1033.3	48.0 (20.3, 66.1)	34	62	6001.4	5967.8	566.5	1038.9	45.4 (15.8, 64.6)
24–35 months	13	6	2637.5	2578.6	492.9	232.7	−114.0 (−463.0, 18.7)	13	7	2670.3	2636.2	486.8	265.5	−83.3 (−359.1, 26.8)
≥ 36 months	0	0	791518.0	788879.0	0.0	0.0	U	0	1	803565.0	808797.0	0.0	0.1	100 (−3774.3, 100)
**Pre-/post-booster**														
Pre-booster	24	47	4033.0	4007.9	595.1	1172.7	49.3 (17.1, 69.0)	26	48	4050.2	4039.4	641.9	1188.3	46.0 (10.9, 67.3)
Post-booster	19	20	5363.8	5263.2	354.2	380.0	6.0 (−76.2, 49.8)	19	20	5425.1	5373.4	350.2	372.2	5.8 (−76.0, 49.6)

AOM, acute otitis media; B-AOM, bacteriologically-confirmed AOM; CI, confidence interval; post-booster, at the time of or after booster vaccination; U, undefined; intent-to-treat analysis, follow-up starting at the time of first vaccination; per-protocol analysis, follow-up starting 2 weeks post-dose 3. Vaccine efficacy was calculated as (1 – hazard ratio) x 100 using a Cox (first episodes) or generalized Cox (all episodes) regression model.

a All B-AOM episodes, including recurrent episodes in the same child.

A preliminary descriptive post-hoc analysis suggested higher incidence of C-AOM in children without any influenza or PHiD-CV vaccination (68.8 episodes per 1000 child-years, compared to 38.5 in children who received only PHiD-CV, 35.4 in children who received only influenza vaccination, and 31.7 in children who received PHiD-CV and influenza vaccination).^34^

### Vaccine efficacy against first clinical AOM episodes

Point estimates for vaccine efficacy (VE) against first C-AOM were positive for the younger age groups: 26.9% for the 2–11 months group, 19.7% for the 12–23 months group, and 24.0% for both groups pooled together (i.e. children < 24 months of age), with the lower limit of the 95% CI being above zero for the 2–11 months and <24 months age groups. However, VE declined in older children, with point estimates of −10.5% in the 24–35 months age group and −33.8% in the ≥ 36 months age group (Table 3).

In a post-hoc analysis by sex, the incidence of, and VE against C-AOM seemed to be higher in boys (Table 3).

Although none of the VE estimates for the different AOM endpoints analyzed reached statistical significance, the consistent trend across the different endpoints suggests a positive effect, with VE point estimates of 17.7% against moderate and 32.7% for severe AOM in the analysis based on complete symptom reporting, and 12.2% against mild AOM episodes when assessing episodes with information on at least one AOM symptom (Table 3).

### Vaccine efficacy against microbiologically-confirmed AOM

VE against first B-AOM episodes was 33.6% (95% CI: 3.2, 54.5) (Supplemental Digital Content 1, Fig. 3). Although the number of B-AOM episodes in each age group was small, point estimates for VE against first B-AOM were positive, with lower limits of the 95% CIs above 0, for the separate 2–11 and 12–23 months age groups, as well as for the combined < 24 months age group (Table 4). VE against B-AOM was positive at pre-booster, while the point estimate observed after the booster dose was much lower than the pre-booster vaccination value and its 95% CI included zero (Table 4).

**Figure 3.**
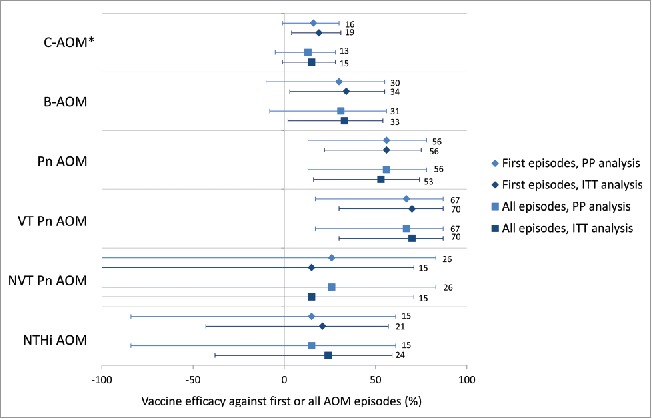
Vaccine efficacy of PHiD-CV against first or all AOM episodes in the per-protocol and intent-to-treat analyses. Footnote: PP: N = 3010 (PHiD-CV); N = 2979 (Control); ITT: N = 3602 (PHiD-CV); N = 3612 (Control). Error bars depict 95% confidence interval; N, number of children in PP or ITT cohort; PP, per-protocol; ITT, intent-to-treat; AOM, acute otitis media; C-AOM, clinically confirmed AOM; B-AOM, bacteriologically confirmed AOM; Pn, *S. pneumoniae*; VT, vaccine type; NVT, non-vaccine non-vaccine-related type; NTHi, non typeable *H. influenzae*. *Vaccine efficacy against first C-AOM in per-protocol analysis was assessed as secondary confirmatory objective.

VE was 55.7% (95% CI: 21.5, 75.0) against first episodes of pneumococcal AOM, and 69.9% (95% CI: 29.8, 87.1) against vaccine-serotype AOM (Supplemental digital content 1, Fig. 3). VE against NTHi AOM could not be demonstrated (VE: 21.5%; 95% CI: −43.4, 57.0) (Supplemental Digital Content 1). There was no evidence of disease replacement of vaccine serotypes by other pneumococcal serotypes (Fig. 3) or by other bacterial pathogens (Table 2). Results for individual vaccine and vaccine-related serotypes are shown in Supplemental Digital content 2.

### Nasopharyngeal colonization with S. pneumoniae and H. influenzae

**Figure 4.**
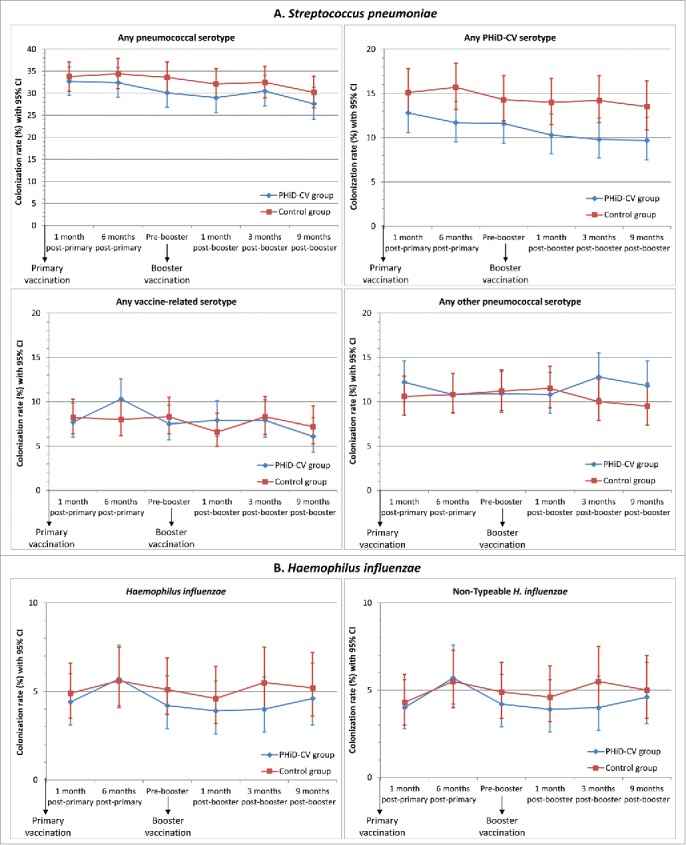
Nasopharyngeal carriage rates of *S. pneumoniae* and *H. influenzae* in nasopharyngeal swabs following primary and booster vaccination with PHiD-CV or control vaccine (intent-to-treat cohort). Footnote: Error bars: 95% confidence intervals. Any serotype belonging to the same serogroup as the PHiD-CV vaccine serotypes, but different from the vaccine serotypes, was considered as vaccine-related. The observed vaccine-related serotypes were 6A, 6C, 19A, and 23A.

A reduction in the carriage of vaccine pneumococcal serotypes was observed after primary and booster vaccination (Fig. 4A). VE against carriage of any vaccine pneumococcal serotype reached 25.6% (95% CI: 1.7, 43.8) at 6 months post-primary vaccination and 31.2% (95% CI: 5.3, 50.3) at 3 months post-booster vaccination; at 9 months post-booster, VE was 27.7% (95% CI: −1.5, 48.8) (Supplemental Digital Content 3).

The most common PHiD-CV serotypes detected at the different time points were serotypes 6B (range, 1.9–5.1%), 19F (range, 2.7–4.8%) and 23F (range, 2.1–4.7%) (Supplemental Digital Content 3). The prevalence of each of the 7 other vaccine serotypes was below 1.0% in the PHiD-CV group and below 1.5% each in the Control group at each time point. There was no evidence of an impact on carriage of vaccine-related serotypes; VE against carriage of any vaccine-related pneumococcal serotype ranged from −27.9% to 15.8% at the different timepoints, and was 2.0% across all time points. The prevalence of vaccine-related serotypes ranged from 2.4% to 5.2% for serotype 6A, from 1.1% to 2.2% for serotype 19A, and from 0.9% to 1.9% for serotype 23A (Fig. 4A, Supplemental Digital Content 3).

Rises in non-vaccine-type nasopharyngeal colonization in the PHiD-CV group were only observed at the latest time points (VE: −27.9% at 3 months post-booster and −23.6% at 9 months post-booster); no higher non-vaccine, non-vaccine-related serotype colonization rate was observed in the PHiD-CV group versus the Control group up to 1 month post-booster (Fig. 4A, Supplemental Digital Content 3). This delayed onset of rises in non-vaccine, non-vaccine-related serotype colonization combined with the decrease in vaccine-type colonization resulted in a trend for a lower rate of colonization by any pneumococcal type in the PHiD-CV vs. Control group at each time point (Fig. 4A, Supplemental Digital Content 3).

The *H. influenzae* colonization rate was very low (range, 4.0–5.7%) at each time point (Fig. 4B), and at least 98% of *H. influenzae* detected were NTHi. The presented data only include results from samples confirmed as positive for *H. influenzae* or NTHi after differentiation from *H. haemolyticus* by polymerase chain reaction (PCR) assay; at least 93% of the cultured NTHi samples were confirmed to be NTHi after PCR differentiation. We did not observe a significant reduction of nasopharyngeal NTHi carriage at any point, with the highest VE being 27.0% (95% CI: −22.2, 56.8) at 3 months following booster vaccination; VE across all time points was 7.4% (95% CI: −15.1, 25.5) (Fig. 4B, Supplemental digital content 3).

### Nasopharyngeal colonization with other bacteria

In addition to *S. pneumoniae*, which were the most commonly isolated bacteria (present in approximately 30% of samples at each time point), and *H. influenzae* which had a markedly lower prevalence (<6%), we also assessed the colonization with other bacteria including *S. aureus, S. pyogenes* and *M. catarrhalis*. Of these other bacteria, *S. aureus* was the most commonly isolated pathogen in both groups (approximately 8%). The colonization rate at 9 months post-booster was 6.5% (95% CI: 4.7, 8.8) in the PHiD-CV group and 6.3% (95% CI: 4.5, 8.4) in the Control group (Supplemental digital content 3). No evidence of an increase in rate of *S. aureus* carriage in the PHiD-CV group vs. the Control group during the study was found. The percentage of nasopharyngeal samples with *S. pyogenes* or *M. catarrhalis* colonization was ≤0.8% in both groups at each time point.

### Acquisition of bacterial nasopharyngeal carriage

Across all sampling time points, vaccination with PHiD-CV was associated with reduced acquisition of vaccine pneumococcal serotypes compared with the control vaccine (Supplemental digital content 4). Acquisition rates for vaccine-related serotypes or other pneumococcal serotypes were within the same ranges in the PHiD-CV and Control groups. Acquisition of *H. influenzae* (all NTHi in the PHiD-CV group, 99% NTHi in the Control group) tended to be lower in the PHiD-CV group than in the Control group. The overall rates of *S. aureus, S. pyogenes* and *M. catarrhalis* acquisition were within the same ranges in the PHiD-CV and Control group.

## Discussion

In this study in more than 7,000 children in Panama, PHiD-CV showed VE against AOM in children younger than 24 months. However, VE declined with age. Our data also suggested a trend towards efficacy against AOM with different levels of severity. Because most cases of AOM were first episodes according to our study definition, the trial can be considered as a study of first AOM episodes. Additionally, a consistent trend for reduction of vaccine-serotype nasopharyngeal carriage was observed in PHiD-CV recipients compared with the Control group.

We previously reported VE against first C-AOM episodes of 16.1% (95% CI: −1.1, 30.4) in the per-protocol analysis and 19.0% (95% CI: 4.4, 31.4) in the intent-to-treat analysis (Fig. 3, Supplemental Digital Content 1).^6^ In the current manuscript, the more granular results of PHiD-CV VE against C-AOM and B-AOM in the intent-to-treat cohort, are presented.

Despite the introduction of an enhanced surveillance method approximately 2 y after the start of the study (Supplemental digital content 5), the total number of reported AOM episodes was lower than could be expected based on incidences in other countries, indicating that our study probably underestimates the true AOM burden. However, the fact that most AOM episodes (≥ 90%) were mild or moderate in severity indicates that there was no substantial bias towards detecting only severe AOM episodes.

Various factors might have contributed to the low incidence of AOM in this study, including antibiotic use, low frequencies of smoking among parents, and high breastfeeding rates.,^24,35^ In addition, high coverage of influenza vaccination in the study population (> 90%) may have lowered the AOM incidence; the incidence of C-AOM appeared to be higher in children without any influenza or PHiD-CV vaccination, in a preliminary descriptive post-hoc analysis.^34^ The complexity of the study procedures such as case ascertainment by both a PHCP and ENT specialist may have played a role in the lower than expected number of AOM cases captured in this study. Moreover, most AOM cases were first episodes (88%), which may be due to a true low percentage of recurrent cases in Panama, reluctance of the parents to bring their child to the clinic for subsequent episodes due to complex study procedures, or a combination of both. In attempt to determine whether caregivers view AOM as a mild condition not generally requiring medical attention, we conducted a subsequent survey at the same health centers. However, according to the survey, caregivers stated that indeed they sought medical care when the child exhibited symptoms that appear to them to be related to ear infections.^36^

VE against C-AOM was mainly driven by VE against moderate and severe episodes (even though CIs span 0); no VE against mild episodes was observed when assessing only episodes with complete positive and negative reporting, on all 5 symptoms, though an indication of such VE was seen when incompletely documented cases were included. We suggest it is reasonable to assume that for cases with missing reports for one or more symptoms, the physician did not document the symptom(s) because they were not present during examination. This could explain why nearly 30% of mild C-AOM cases could not be included in the analysis of severity with complete reporting (vs. 13% of moderate and 6% of severe episodes).

We note that the proportion of MEF samples with culture positivity was low, and the bacterial etiology of AOM could only be confirmed for a limited number of episodes. This may have been due to antibiotic treatment before MEF collection. We did not find evidence for laboratory issues or problems with transport of the samples that could explain the low culture positivity rates.

In the present study, PHiD-CV VE against both C-AOM and B-AOM was observed in children below 24 months of age, but appeared to decrease after the second year of life. The fact that no (C-AOM) or limited (B-AOM) evidence of VE was observed following the booster vaccination was unexpected, since robust immune responses were induced by the booster dose in the present study, as described previously.^6^ However, waning efficacy against AOM with age has previously also been reported with 7vCRM in the Northern California Kaiser Permanente trial, in which children received a booster dose at 12 to 15 months of age and were followed-up to 3.5 y of life.^21^ In contrast, no decrease in 7vCRM efficacy with age was reported in the FinOM trial, in which information was obtained via interviews of parents of children aged 4–5 y.^19^ However, the lack of waning VE reported in the FinOM trial should be interpreted cautiously because parents were aware of the vaccination status of their child, which could have affected their care-seeking behavior.^24^ The lower VE against AOM observed in older children despite the PHiD-CV booster dose may be due to the fact that AOM incidences start decreasing naturally after 18 months of age, and thus, children who continue to have AOM beyond that age could be otitis-prone children.

In the current study, VE against World Health Organization-defined consolidated community-acquired pneumonia (CAP) remained above 20% among children aged 12–24 months and 24–36 months.^6^ This observation may indicate that a specific change in the etiology of AOM among older children, rather than a generic loss of VE against mucosal diseases with age, is responsible for the waning VE against AOM. We did not have enough cases to robustly analyze the etiology of AOM in older children, but other studies have shown the importance of other pathogens including *Streptococcus pyogenes, Moraxella catarrhalis* and *Staphylococcus aureus* as bacterial agents that cause AOM.^11,12^

In PMS studies, decreases in AOM incidence have been observed after introduction of PCV13 and PHiD-CV. When compared to the 7vCRM period, a 19% reduction in OM was observed after introduction of PCV13 in the UK,^17^ while in Israel, a 43% decrease in all-cause AOM was observed.^18^ In children younger than 2 y in the US, OM visit rates had been decreasing annually at a rate of 0.03/ child-year during 2001–2009, but a more prominent decrease of 0.27/child-year was observed in 2010–2011, coinciding with PCV13 introduction.^16^ For PHiD-CV, a significant reduction in children's hospital visits due to AOM was observed in Iceland when comparing the 3-year period before immunization (2008–2010) with the 3 y after PHiD-CV initiation (2011–2013; incidence rate ratio: 0.76).^37^ In indigenous children in Australia, children vaccinated with PHiD-CV had less suppurative OM than children vaccinated with 7vCRM, while a concomitant increase in OM with effusion was observed. Such a shift from suppurative to non-suppurative disease may decrease antibiotic prescription rates and may change demand for surgical procedures (e.g., tympanoplasty, tympanostomy tubes).^38^ However, findings from these PMS studies cannot readily be compared with those from double-blind RCTs. RCTs assess the vaccine under strictly controlled conditions, while PMS studies evaluate the effectiveness in real-life settings but are consequently subject to confounding factors. AOM is an especially difficult end point to assess in PMS studies; PMS results for 7vCRM impact on AOM varied widely, and were clearly confounded by local variability in baseline incidence and in viral and bacterial etiology, lack of a strict case definition, and changes in other societal factors that impact AOM (e.g., household smoking, changes in antibiotic practices).^24^ Because no double-blind RCTs assessing PCV13 impact on AOM are available, we could not directly compare our findings with PCV13.

**Figure 5.**
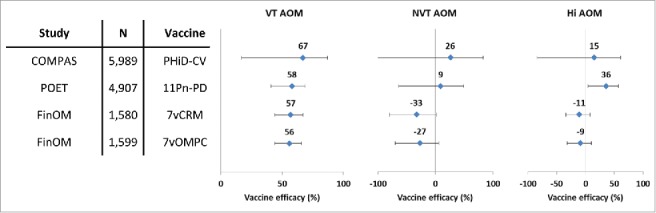
Vaccine efficacy of different pneumococcal conjugate vaccines against all AOM episodes in double-blind randomized controlled trials (per-protocol analyses). Footnote: Error bars: 95% confidence intervals; AOM, acute otitis media; COMPAS, Clinical Otitis Media and PneumoniA Study; POET, Pneumococcal Otitis Efficacy Trial; FinOM, Finnish Otitis Media trial; N, number of children in the per-protocol cohort; VT, vaccine type; NVT, non-vaccine non-vaccine-related type; Hi, H. influenzae; 11Pn-PD, 11-valent protein D-conjugated PCV (unlicensed); 7vCRM, 7-valent CRM197-conjugated PCV (licensed); 7vOMPC, 7-valent meningococcal outer membrane protein complex-conjugated PCV (unlicensed).

VE against AOM episodes caused by vaccine pneumococcal serotypes was 70% in the intent-to-treat analysis and 67% in the per-protocol analysis, which was in line with the results of a previous trial conducted in Finland with 7vCRM^14^ and an investigational 7-valent PCV using meningococcal outer membrane protein complex as carrier protein (Fig. 5).^39^ The results of the present study were also consistent with those of the Pneumococcal Otitis Efficacy Trial (POET), where VE of the 11-valent predecessor to PHiD-CV was 65.5% against AOM episodes due to all vaccine-related serotypes combined during the per-protocol follow-up that started 2 weeks after the third vaccine dose and continued up to 24–27 months of age.^40^ Despite differences in study design, case ascertainment and case definitions, the similarity of the observed VE against AOM due to pneumococcal serotypes contained in the respective PCVs was remarkable. In the present study, no evidence of disease replacement of vaccine serotypes by other serotypes was found, and the number of AOM episodes caused by non-vaccine serotypes was small.

The nasopharyngeal colonization rate with vaccine pneumococcal serotypes was consistently lower after vaccination with PHiD-CV in comparison with the control vaccine in the present study, while acquisition of any vaccine-related serotype, any other non-vaccine, non-vaccine-related serotype, or *S. aureus* was generally consistent between groups. Nevertheless, an increase in carriage of non-vaccine, non-vaccine-related serotypes was observed after the PHiD-CV booster dose, suggesting that some pneumococcal serotype replacement may occur in the niche created by vaccine-type nasopharyngeal carriage reduction. This rise could not easily be attributed to any specific non-vaccine serotype, as carriage rates for individual non-vaccine, non-vaccine-related serotypes did not exceed 2.3% at any time point (data not shown). The examination of individual serotypes showed that the observed increase in the PHiD-CV group was caused by additional isolates for a number of serotypes. This tendency suggests that caution should be exercised in assuming that serotype replacement may not expand over long-term periods.

Nevertheless, the fact that trends for elevated carriage of non-vaccine pneumococcal serotypes were only seen at 3 and 9 months post-booster, long after the decrease in vaccine-type carriage was apparent, contrasts with the results of randomized clinical trials and post-marketing studies showing that 7vCRM vaccination tended to be associated with serotype replacement concurrent with or only slightly delayed after vaccine-type decreases.^23^ The reduction in nasopharyngeal carriage of vaccine pneumococcal serotypes following PHiD-CV vaccination tended to be lower in the present study than in previous studies on PHiD-CV^15,41^ or on other PCVs that were administered in the same age group.^25-27,42-46^ The impact of 7vCRM on vaccine-type nasopharyngeal carriage has been reported to be around 43%,^47,^^48^ and reductions in vaccine-type carriage (mainly the 6 new vaccine serotypes) were also observed after PCV13 introduction, both in general,^49,^^50^ and in children with AOM.^51,52^ Some studies also show a modest decrease in overall pneumococcal carriage after PCV13 introduction, despite the increase in non-vaccine-serotype carriage,^53,54^ while other reports indicate stable overall pneumococcal carriage rates after PCV introduction.^49,55,56^ However, comparisons among carriage trials are difficult due to the influence of a variety of factors on pneumococcal nasopharyngeal carriage, such as geographical location, use of day-care centers, number of siblings, passive smoking, antibiotic pressure, seasonality and circulation of individual serotypes.^57,58^

A dynamic model of pneumococcal infection predicted that vaccines with relatively small effectiveness against colonization by vaccine serotypes could have a major impact on the incidence of invasive pneumococcal disease (IPD) caused by those serotypes in the target population.^59^ A reduction of colonization by 20–30% was identified as a threshold beyond which any PCV with higher VE would have only a marginal incremental benefit on IPD incidence. The magnitude of the effect on AOM caused by vaccine pneumococcal serotypes was similar to that seen with 7vCRM, despite apparent differences in the impact on nasopharyngeal carriage of vaccine serotypes between the 2 vaccines. This observation suggests that a similar colonization threshold may exist for AOM protection and indicates that there is no close quantitative relationship between the magnitude of the impact on carriage and on AOM.^40^

The reductions of vaccine pneumococcal serotype carriage seen in this study will likely have clinical significance, as reductions with other PCVs have been closely associated with substantial herd protection of unvaccinated individuals.^60-64^ Decreased transmission was directly demonstrated in a randomized controlled double-blind clinical trial with PHiD-CV in Finland, where a 29% reduction in vaccine serotype nasopharyngeal and oropharyngeal carriage was observed among unvaccinated older children whose younger siblings had received PHiD-CV.^63^ Consistent with this finding, vaccine-serotype nasopharyngeal carriage was also lower in vaccine-ineligible age groups in Kenya within 2 y after introduction of PHiD-CV in the national infant vaccination program.^62^ Importantly, reductions in IPD rates were also observed in vaccine-ineligible age groups within 1 to 3 y after PHiD-CV implementation in vaccination programs in Finland,^64,65^ Iceland^61^ and Brazil^60^

While an association has been made between pneumococcal disease and nasopharyngeal carriage of *S. pneumoniae*,^31,66,67^ the relationship is not well established for disease caused by other bacteria, including NTHi. In the present study, no significant decrease of NTHi colonization was observed at any point. Based on our findings and those from previous studies,^15,68,69^ it appears that even if PHiD-CV induces a decrease in NTHi carriage, it is subtle and transient.

It is difficult to conclude on the clinical relevance of the limited (or no) effect of vaccination on overall NTHi carriage, especially if only a pathogenic subset of NTHi is actually responsible for clinical AOM.^70^ By analogy, if one had only considered 7vCRM effects on overall pneumococcal colonization, with no net decrease, one might have concluded that the vaccine would not affect pneumococcal AOM; instead, it was necessary to look specifically at the vaccine-type carriage and vaccine-type pneumococcal disease.

This is the first study reporting simultaneous assessment of the effects of PHiD-CV on both carriage and AOM with assessment of bacterial etiology. However, this study has a number of limitations. First, the study was limited by the low number of reported AOM and of bacteriologically confirmed cases. Moreover, the results of the descriptive analyses presented in this manuscript should be interpreted with caution because there was no adjustment for multiplicity and no pre-defined success criteria were set. Finally, the interpretation of the descriptive analyses of VE per age subgroup and before vs. after booster dose was limited by the difficulty of separating the effect of age from the effect of the booster dose.

In conclusion, PHiD-CV showed efficacy against AOM in children aged less than 24 months, who are the most vulnerable population for AOM and its consequences. Positive VE estimates were observed against a range of AOM severity categories up to the booster dose administration, indicating that the results from this trial are likely relevant to the commonly seen clinical range of AOM cases and that PHiD-CV vaccination may have a considerable impact on public health. The decreases in AOM were accompanied by significant decreases in vaccine-serotype nasopharyngeal colonization with only a small, delayed tendency for increases in colonization with non-vaccine-type pneumococci and no evidence of changes in carriage of other pathogens. These findings are consistent with emerging evidence of herd protection against disease in unvaccinated age groups in countries using PHiD-CV.

## Materials and methods

### Study design and participants

COMPAS was a phase III, double-blind, randomized controlled study conducted in Argentina, Colombia and Panama (ClinicalTrials.gov: NCT00466947). Its primary objective was to demonstrate PHiD-CV efficacy against first episodes of likely bacterial community-acquired pneumonia (B-CAP) in infants and young children. The study objectives and key efficacy results for CAP, AOM and IPD have already been reported.^6^ AOM and nasopharyngeal carriage were only assessed in Panama, and were considered as secondary outcomes of the COMPAS study.

In Panama, the study was conducted between August 2007 and July 2011 in 16 centers. A total of 7,359 children were randomized (1:1 ratio) to receive either PHiD-CV (PHiD-CV group) or control vaccines (Control group) at 2, 4, 6 and 15–18 months of age, as described previously.^6^ In short, the PHiD-CV group received PHiD-CV (*Synflorix™*) and diphtheria–tetanus–acellular pertussis–hepatitis B–inactivated poliovirus–*Haemophilus influenzae* type b vaccine (*Infanrix hexa™*) at 2, 4, and 6 months of age followed by one dose of PHiD-CV at 15–18 months of age, while the control group received hepatitis B vaccine (*Engerix-B™*) and diphtheria–tetanus–acellular pertussis–inactivated poliovirus–*Haemophilus influenzae* type b vaccine (DTPa-IPV/Hib; *Infanrix™-IPV/Hib*) followed by one dose of hepatitis A vaccine (*Havrix™*).

Written informed consent was obtained from children's parents or guardians. The study was conducted in accordance with Good Clinical Practice, all applicable regulatory requirements and the Declaration of Helsinki, except for the deviations that were described previously,^6^ and are also detailed in the text of Supplemental Digital Content 6. The full protocol has been previously published.^6^

### Assessment of AOM

**Figure 6.**
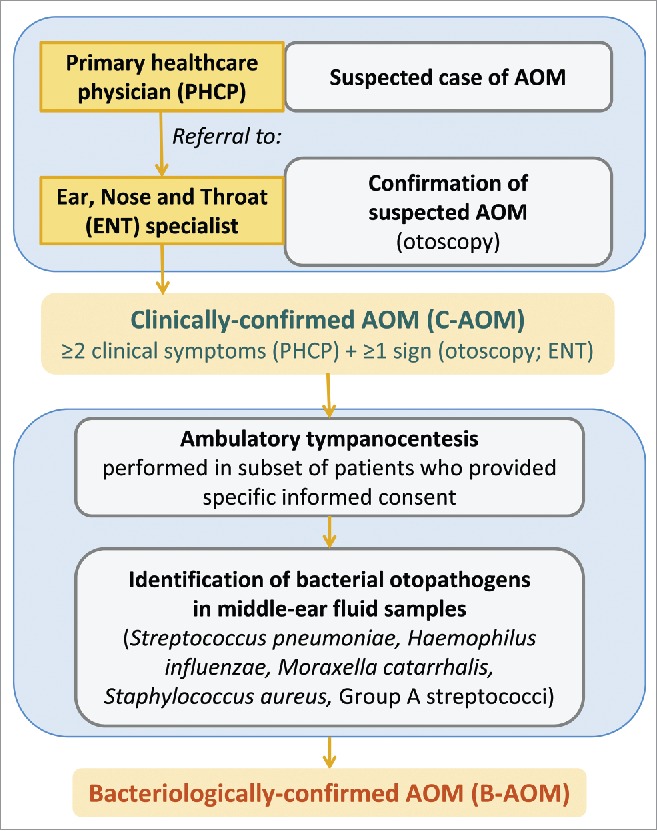
AOM surveillance and definition of clinically-confirmed AOM (C-AOM) and bacteriologically-confirmed AOM (B-AOM). AOM, acute otitis media.

The methodology for the surveillance of AOM was described previously,^6^ and is summarized in the text of Supplemental Digital Content 5 and Fig. 6. All children were followed-up for AOM, and all otitis media visits were reported.

If AOM was suspected by a PHCP, the child was referred to an ENT specialist involved in the trial. The diagnosis of C-AOM required at least 2 clinical symptoms (ear pain, ear discharge, hearing loss, fever, lethargy, irritability, anorexia, vomiting or diarrhea) which had started within the previous 5 d and were reported by the PHCP; and either altered visual appearance of the tympanic membrane (e.g., redness, bulging, loss of light reflex) or the presence of middle ear effusion as observed by the ENT specialist.

MEF sampling through tympanocentesis was proposed to children with a C-AOM that was confirmed by an ENT specialist. Initially, if the severity according to the OS-8 scale^71^ was less than 3, the recommendation was not to perform tympanocentesis. Later in the study, in an attempt to obtain more samples, MEF samples were collected whenever the ENT specialists considered that tympanocentesis needed to be performed (i.e., when liquid was present behind the ear drum, even in a patient with OS score less than 3). A sub-analysis comparing results in the 2 periods was not performed, because not enough samples were available to allow a meaningful analysis. Approximately 9% of MEF samples were collected from spontaneous drainage. If a child had already been treated with antibiotics by pediatricians, this fact was recorded in the child's study file and tympanocentesis was performed in children who had been receiving antibiotics for < 72 hours, or for children treated for > 72 hours with antibiotics other than amoxicillin, amoxicillin-clavulanate, or ceftriaxone. Before MEF sampling, additional written informed consent was obtained from parents or guardians of all children, except for a few cases described in the text of Supplemental Digital Content 6.

An C-AOM was considered of bacterial etiology (B-AOM) if a bacterial pathogen recognized as causative of AOM (such as *H. influenzae, S. pneumoniae, Staphylococcus aureus, Streptococcus pyogenes* or *Moraxella catarrhalis*) was identified in the MEF sample using standard microbiological culture methods (see Supplemental Digital Content 7).

For purpose of the analyses, a new AOM episode was defined when ≥ 30 d elapsed from the start of a previous AOM episode (according to the date confirmed by the ENT specialist). If an AOM case was reported within 30 d from the start date of a previous episode, it was considered as the same clinical episode. A new B-AOM episode was defined whenever a different bacterial pathogen was isolated at the latter visit. First AOM episodes were defined as the first episode reported for this child in the course of the study.

The clinical/otologic scale developed by Dagan *et al.* was used to assess the severity of each suspected AOM case.^33^ This scale is based on 5 symptoms: temperature, irritability and ear tugging on the day of the visit as reported by the parents, and redness and bulging of the tympanic membrane, as observed at otoscopy performed by the ENT. A total score of 0–4 was considered indicative of mild AOM, 5–7 of moderate AOM, and 8–15 of severe AOM (Table 5).

**Table 5.** Clinical/otologic scale developed by Dagan et al. to assess the severity of AOM episodes.^24^

Score	Temperature^a^	Irritability^a^	Tugging^a^	Redness^b^	Bulging^b^
0	<38.0°C	Absent	Absent	Absent	Absent
1	38.0–38.5°C	Mild	Mild	Mild	Mild
2	38.6–39.0°C	Moderate	Moderate	Moderate	Moderate
3	>39.0°C	Severe	Severe	Severe^c^	Severe^c^

a Reported by parents on the day of the visit.

b Observed at the otologic examination.

c Including spontaneous drainage of pus.

### Assessment of nasopharyngeal carriage

The impact of PHiD-CV on nasopharyngeal carriage was assessed in a subset of the AOM efficacy cohort, consisting of 2,000 children (carriage subset). Nasopharyngeal swabs were taken at 6 time points: at 7 months of age (1 month after primary vaccination), 12–15 months of age (approximately 6–9 months after primary vaccination), before booster vaccination (15–18 months of age), and 1, 3 and 9 months after the booster dose (Fig. 2).

Nasopharyngeal swabs were collected by study personnel using pediatric rayon-tipped swabs with flexible aluminum shaft, although non-flexible swabs were also used in approximately 200 samples because of temporary unavailability of flexible swabs at some centers. Microbiological procedures are detailed in the text of Supplemental Digital Content 7.

## Statistical analysis

### AOM

The determination of the sample size and study power, the definitions of the study cohorts, and the calculation method for VE were described previously^6^ and are detailed in text of Supplemental Digital Content 8.

PHiD-CV VE against first C-AOM episodes occurring in the per-protocol cohort as of 2 weeks after dose 3 was considered significant if the primary study objective to demonstrate VE against first B-CAP episodes was significant and the one-sided P-value for VE against C-AOM was < 0.025; the results of these confirmatory objectives were reported in the primary manuscript.^6^ The current manuscript reports secondary objectives assessed in a descriptive manner. Pre-defined secondary AOM endpoints including the evaluation of AOM episodes caused by *S. pneumoniae* and NTHi, were descriptive with no adjustment for multiplicity. No pre-defined success criteria were fixed but from a descriptive point of view, conclusion on statistical significance of VE was based on a positive lower limit of its non-adjusted 95% CI and statistically significant differences between groups were defined based on non-overlapping 95% CI boundaries around incidence rates. However, no strict statistical conclusions can be made in these kinds of exploratory analyses; the focus is on trends.

PHiD-CV VE against AOM was further characterized descriptively (without pre-defined statistical criteria) by age (2–11 months, 12–23 months, both age groups < 24 months pooled together, 24–35 months, and ≥ 36 months), before vs. after booster vaccination (taking only the pre- and post-booster AOM cases into account for children who received all 4 vaccine doses), and by severity (for AOM episodes that could be graded for severity based on the complete or partial reporting of symptoms). AOM severity was categorized according to the 5 symptoms used in the clinical/otologic scale developed by Dagan *et al*, as described above.^33^ We performed a first analysis including only AOM episodes for which the presence or absence of each of the 5 symptoms had been recorded; and a second analysis including all AOM episodes with at least one symptom score recorded, assuming that unrecorded symptoms were not observed.

For the analysis according to AOM severity, different levels of severity for the same AOM episode (i.e., occurring within 30 days) were counted as individual endpoints and analyzed as separate AOM episodes (for example, if a child presented with mild AOM, and 1 week later with severe AOM, this was considered to be one AOM episode, but contributed once to the analysis of mild AOM cases and once to the analysis of severe AOM cases). As a consequence, the sum of all mild, moderate and severe episodes could be higher than the total number of AOM cases counted when not considering the severity. Cases of the same severity level occurring within 30 d (for example, 2 mild episodes) were counted as 1 episode. The same methodology was applied for the analysis of AOM episodes reported before and after the booster dose. For the analysis by age, the total number of reported AOM episodes was counted considering the appropriate cohort and follow-up, and the 30-day rule was applied for the analysis of all episodes. Episodes were then subdivided per defined age range according to the age of children at the time of the considered episode. The sum of episodes in the various categories was thus equal to the total number of episodes.

### Nasopharyngeal carriage

Percentages of children with nasopharyngeal swabs positive for *S. pneumoniae, H. influenzae* or other bacterial pathogens at each swab time point, as well as the frequency of acquisition of new bacteria or serotypes were calculated with non-adjusted 95% CIs. A new acquisition at a particular visit was defined when a child's nasopharyngeal swab became positive for a specific bacterium or serotype after being negative at the previous visit for that specific bacterium or serotype.

In the intent-to-treat cohort, PHiD-CV VE against nasopharyngeal carriage of bacterial pathogens was estimated as 1 minus the relative risk. Statistical significance of VE was assessed based on a lower limit of the 95% CI around VE >0 with no adjustment for multiplicity.

## Trademark statement

Prevenar/Prevnar and Prevenar 13/Prevnar 13 are trademarks of Wyeth Pharmaceuticals Inc., a subsidiary of Pfizer Inc. Synflorix, Infanrix, Infanrix hexa, Engerix-B, and Havrix are trademarks of the GSK group of companies.

## Supplementary Material

Supplemental_Material.zip

## Abbreviations

7vCRM: 7-valent CRM197-conjugate vaccine
AOM: acute otitis media
B-AOM: bacteriologically-confirmed AOM
B-CAP: likely bacterial community-acquired pneumonia
C-AOM: clinically-confirmed AOM
CAP: community-acquired pneumonia
CI: confidence interval
COMPAS: Clinical Otitis Media and Pneumonia Study
ENTear: earnose and throat
FinOM: Finnish Otitis Media trial
IPD: invasive pneumococcal disease
MEF: middle ear fluid
NPC: nasopharyngeal carriage
NTHi: non-typeable *Haemophilus influenzae*
PCR: polymerase chain reaction
PCV: pneumococcal conjugate vaccine
PHCP: primary healthcare physician
PHiD-CV: 10-valent pneumococcal non-typeable *Haemophilus influenzae* protein D conjugate vaccine
POET: Pneumococcal Otitis Efficacy Trial
VE: vaccine efficacy
