# Supplementary material for: Efficacy of 10-valent pneumococcal non-typeable Haemophilus influenzae protein D conjugate vaccine against acute otitis media and nasopharyngeal carriage in Panamanian children – A randomized controlled trial
Source: Hum Vaccin Immunother. 2017 Feb 25;13(6):1213–28. doi: 10.1080/21645515.2017.1287640 (PMC5489287; doi:10.1080/21645515.2017.1287640)
Supplement: Supplemental_Material.zip [file khvi-13-06-1287640-s001.zip › Supplemental digital content 9.docx]

**Supplemental digital content 9.** Sequences and final concentrations of the oligonucleotides used in the duplex *lgtC*/*P6* real-time PCR used to distinguish *H. influenzae* from *H. haemolyticus* strains.

| **Oligonucleotide** | **Sequence** | **Modification** | **Final concentration (nM)** | **Provider** |
| --- | --- | --- | --- | --- |
| LgtC Reverse primer | 5’-TATCTCGACATSCTGCCAAATAATA-3’ | N/A | 400 | Eurogentec |
| LgtC Forward primer | 5’-TRGCKACTTACGCTAGACTAAATTTAACTAA-3’ | N/A | 400 | Eurogentec |
| LgtC TaqMan^®^ | 5’-ACATTAACAAACTCTTCACTTC-3’ | 5’ FAM | 200 | Applied Biosystems |
| MGB Probe |  | 3’ NFQ-MGB |  |  |
| P6 Forward primer | 5’-TCACCGTAAGATACTGYGCCTAATTT-3’ | N/A | 200 | Eurogentec |
| P6 Reverse primer | 5’-GTACACCAGAATACAACATCGCATT-3’ | N/A | 200 | Eurogentec |
| P6 TaqMan^®^ | 5‘-TAAATAACCTTTAACTGCATCTG-3’ | 5’ VIC^TM^ | 200 | Applied Biosystems |
| MGB Probe |  | 3’ NFQ-MGB |  |  |

N/A, not applicable; PCR, polymerase chain reaction
