# Supplementary material for: Efficacy of 10-valent pneumococcal non-typeable Haemophilus influenzae protein D conjugate vaccine against acute otitis media and nasopharyngeal carriage in Panamanian children – A randomized controlled trial
Source: Hum Vaccin Immunother. 2017 Feb 25;13(6):1213–28. doi: 10.1080/21645515.2017.1287640 (PMC5489287; doi:10.1080/21645515.2017.1287640)
Supplement: Supplemental_Material.zip [file khvi-13-06-1287640-s001.zip › Supplemental digital content 1 .docx]

**Supplemental digital content 1.** Vaccine efficacy of PHiD-CV against first or all AOM episodes.

|  | **Intent-to-treat analysis** | | **Per-protocol analysis** | |
| --- | --- | --- | --- | --- |
| **Case definition** | **VE against first AOM episodes, % (95% CI)** | **VE against all AOM episodes, % (95% CI)** | **VE against first AOM episodes, % (95% CI)** | **VE against all AOM episodes, % (95% CI)** |
| C-AOM | 19.0 (4.4, 31.4) | 14.8 (-1.0, 28.2) | 16.1 (-1.1, 30.4) | 12.8 (-5.3, 27.8) |
| B-AOM | 33.6 (3.2, 54.5) | 33.3 (2.3, 54.5) | 29.9 (-10.4, 55.4) | 31.0 (-8.4, 56.1) |
| Pneumococcal AOM | 55.7 (21.5, 75.0) | 52.9 (16.0, 73.6) | 56.1 (13.4, 77.8) | 55.9 (13.2, 77.6) |
| Vaccine serotype AOM | 69.9 (29.8, 87.1) | 69.8 (29.7, 87.0) | 67.1 (17.0, 86.9) | 67.0 (16.9, 86.9) |
| Vaccine-related serotypes^a^ | 29.0 (-123.7, 77.5) | 29.0 (-123.4, 77.4) | 25.7 (-232.2, 83.4) | 25.6 (-232.3, 83.4) |
| Other serotype AOM | 14.8 (-153.7, 71.4) | 14.7 (-153.3, 71.3) | 25.7 (-231.9, 83.4) | 25.7 (-231.2, 83.3) |
| *H. influenzae* AOM | 17.3 (-49.8, 54.3) | 20.5 (-44.3, 56.2) | 15.0 (-83.8, 60.7) | 15.0 (-83.6, 60.6) |
| NTHi AOM | 21.5 (-43.4, 57.0) | 24.5 (-38.3, 58.8) | 15.0 (-83.8, 60.7) | 15.0 (-83.6, 60.6) |

AOM, acute otitis media; C-AOM, clinically-confirmed AOM; B-AOM, bacteriologically-confirmed AOM; NTHi, non-typeable *Haemophilus influenzae*; VE, vaccine efficacy; 95% CI, 95% confidence interval; intent-to-treat analysis, follow-up starting at the time of first vaccination; per-protocol analysis, follow-up starting 2 weeks post-dose 3. ^a^Pneumococcal serotype 6A, 18B, 19A or 23A.
