# Supplementary material for: Efficacy of 10-valent pneumococcal non-typeable Haemophilus influenzae protein D conjugate vaccine against acute otitis media and nasopharyngeal carriage in Panamanian children – A randomized controlled trial
Source: Hum Vaccin Immunother. 2017 Feb 25;13(6):1213–28. doi: 10.1080/21645515.2017.1287640 (PMC5489287; doi:10.1080/21645515.2017.1287640)
Supplement: Supplemental_Material.zip [file khvi-13-06-1287640-s001.zip › Supplemental digital content 2.docx]

**Supplemental digital content 2.** Serotype identification in MEF samples of episodes of pneumococcal AOM anytime during the trial (Total vaccinated cohort)

|  | **PHiD-CV  N = 3602** | | | | **Control  N = 3612** | | | |
| --- | --- | --- | --- | --- | --- | --- | --- | --- |
|  |  | | **95% CI** | |  | | **95% CI** | |
|  | **n** | **%** | **LL** | **UL** | **n** | **%** | **LL** | **UL** |
| **Any vaccine serotype** | 7 | 0.19 | 0.08 | 0.40 | 23 | 0.64 | 0.40 | 0.95 |
| Serotype 1 | 0 | 0 | 0.00 | 0.10 | 0 | 0 | 0.00 | 0.10 |
| Serotype 4 | 0 | 0 | 0.00 | 0.10 | 1 | 0.03 | 0.00 | 0.15 |
| Serotype 5 | 0 | 0 | 0.00 | 0.10 | 0 | 0 | 0.00 | 0.10 |
| Serotype 6B | 0 | 0 | 0.00 | 0.10 | 4 | 0.11 | 0.03 | 0.28 |
| Serotype 7F | 0 | 0 | 0.00 | 0.10 | 0 | 0 | 0.00 | 0.10 |
| Serotype 9V | 0 | 0 | 0.00 | 0.10 | 1 | 0.03 | 0.00 | 0.15 |
| Serotype 14 | 1 | 0.03 | 0.00 | 0.15 | 3 | 0.08 | 0.02 | 0.24 |
| Serotype 18C | 0 | 0 | 0.00 | 0.10 | 1 | 0.03 | 0.00 | 0.15 |
| Serotype 19F | 5 | 0.14 | 0.05 | 0.32 | 11 | 0.30 | 0.15 | 0.54 |
| Serotype 23F | 1 | 0.03 | 0.00 | 0.15 | 2 | 0.06 | 0.01 | 0.20 |
| **Any vaccine- related serotype** | 5 | 0.14 | 0.05 | 0.32 | 7 | 0.19 | 0.08 | 0.40 |
| Serotype 6A | 1 | 0.03 | 0.00 | 0.15 | 3 | 0.08 | 0.02 | 0.24 |
| Serotype 18B | 0 | 0 | 0.00 | 0.10 | 1 | 0.03 | 0.00 | 0.15 |
| Serotype 19A | 4 | 0.11 | 0.03 | 0.28 | 2 | 0.06 | 0.01 | 0.20 |
| Serotype 23A | 0 | 0 | 0.00 | 0.10 | 1 | 0.03 | 0.00 | 0.15 |

N, number of children in the total vaccinated cohort; n (%), number (percentage) of episodes of the defined type of AOM; CI, confidence interval; LL, lower limit; UL, upper limit.
