# Supplementary material for: Efficacy of 10-valent pneumococcal non-typeable Haemophilus influenzae protein D conjugate vaccine against acute otitis media and nasopharyngeal carriage in Panamanian children – A randomized controlled trial
Source: Hum Vaccin Immunother. 2017 Feb 25;13(6):1213–28. doi: 10.1080/21645515.2017.1287640 (PMC5489287; doi:10.1080/21645515.2017.1287640)
Supplement: Supplemental_Material.zip [file khvi-13-06-1287640-s001.zip › Supplemental digital content 3.docx]

**Supplemental digital content 3.** Nasopharyngeal carriage rates of *S. pneumoniae*, *H. influenzae* or other bacterial pathogens and vaccine efficacy at selected sampling time points: 6 months after primary vaccination, 3 and 9 months after booster vaccination, and across all visits (intent-to-treat cohort).

|  | **6 months post-primary** | | |  | **3 months post-booster** | | |  | **9 months post-booster** | | |  | **Across all visits** | | |
| --- | --- | --- | --- | --- | --- | --- | --- | --- | --- | --- | --- | --- | --- | --- | --- |
|  | **Colonization, % (95% CI)** | | **Vaccine efficacy, % (95% CI)** |  | **Colonization, % (95% CI)** | | **Vaccine efficacy, % (95% CI)** |  | **Colonization, % (95% CI)** | | **Vaccine efficacy, % (95% CI)** |  | **Colonization, % (95% CI)** | | **Vaccine efficacy, % (95% CI)** |
|  | **PHiD-CV  (N = 788^a^)** | **Control (N = 784^b^)** |  |  | **PHiD-CV  (N = 696)** | **Control (N = 690)** |  |  | **PHiD-CV (N = 627^c^)** | **Control (N = 639^d^)** |  |  | **PHiD-CV (N = 852 ^e^)** | **Control (N = 848)** |  |
| **Any pneumococcal serotype** | 32.4  (29.1, 35.8) | 34.4  (31.1, 37.9) | 6.0  (-11.9, 21.1) |  | 30.5  (27.1, 34.0) | 32.5 (29.0, 36.1) | 6.2  (-13.7, 22.6) |  | 27.6  (24.1, 31.3) | 30.2  (26.7, 33.9) | 8.6  (-12.7, 26.0) |  | 67.4 (64.1, 70.5) | 69.5 (66.2, 72.5) | 3.0 (-9.0, 13.7) |
| **Any PHiD-CV serotype** | 11.7  (9.5, 14.1) | 15.7  (13.2, 18.4) | 25.6*****  (1.7, 43.8) |  | 9.8  (7.7, 12.2) | 14.2  (11.7, 17.0) | 31.2*****  (5.3, 50.3) |  | 9.7  (7.5, 12.3) | 13.5  (10.9, 16.4) | 27.7  (-1.5, 48.8) |  | 31.9 (28.8, 35.2) | 42.9 (39.6, 46.3) | 25.6* (12.7, 36.7) |
| **Individual PHiD-CV serotypes** | | | |  |  |  |  |  |  |  |  |  |  |  |  |
| 1 | 0.0  (0.0, 0.5) | 0.0  (0.0, 0.5) | U |  | 0.0  (0.0, 0.5) | 0.1  (0.0, 0.8) | 100 (-3766.4, 100) |  | 0.0  (0.0, 0.6) | 0.0  (0.0, 0.6) | U |  | 0.0  (0.0, 0.4) | 0.1  (0.0, 0.7) | 100 (-3781.7, 100) |
| 4 | 0.0  (0.0, 0.5) | 0.4  (0.1, 1.1) | 100 (-140.8, 100) |  | 0.1  (0.0, 0.8) | 0.0 (0.0, 0.5) | I |  | 0.3  (0.0, 1.1) | 0.3  (0.0, 1.1) | -1.9 (-1306.0, 92.6) |  | 0.7 (0.3, 1.5) | 1.1 (0.5, 2.0) | 33.6 (-108.7, 80.6) |
| 5 | 0.0  (0.0, 0.5) | 0.0  (0.0, 0.5) | U |  | 0.0  (0.0, 0.5) | 0.0  (0.0, 0.5) | U |  | 0.0  (0.0, 0.6) | 0.0  (0.0, 0.6) | U |  | 0.0  (0.0, 0.4) | 0.0  (0.0, 0.4) | U |
| 6B | 3.3  (2.2, 4.8) | 4.7  (3.3, 6.4) | 30.1 (-18.6, 59.4) |  | 1.9  (1.0, 3.2) | 5.1  (3.6, 7.0) | 63.2***** (28.7, 82.1) |  | 2.7  (1.6, 4.3) | 3.3  (2.0, 5.0) | 17.5 (-64.2, 59.1) |  | 8.7 (6.9, 10.8) | 12.9 (10.7, 15.3) | 32.4* (8.4, 50.4) |
| 7F | 0.0  (0.0, 0.5) | 0.0  (0.0, 0.5) | U |  | 0.0  (0.0, 0.5) | 0.1  (0.0, 0.8) | 100 (-3766.4, 100) |  | 0.0  (0.0, 0.6) | 0.2  (0.0, 0.9) | 100 (-3874.6, 100) |  | 0.0  (0.0, 0.4) | 0.2 (0.0, 0.8) | 100 (-430.0, 100) |
| 9V | 0.4  (0.1, 1.1) | 0.0 (0.0, 0.5) | I |  | 0.1  (0.0, 0.8) | 0.1 (0.0, 0.8) | 0.9 (-7682.0, 98.7) |  | 0.2  (0.0, 0.9) | 0.3 (0.0, 1.1) | 49.0 (-878.8, 99.1) |  | 0.9 (0.4, 1.8) | 0.8 (0.3, 1.7) | -13.7 (-268.5, 64.0) |
| 14 | 0.9  (0.4, 1.8) | 0.5 (0.1, 1.3) | -74.1 (-711.1, 55.7) |  | 0.6  (0.2, 1.5) | 1.4 (0.7, 2.6) | 60.3 (-37.5, 90.9) |  | 0.5  (0.1, 1.4) | 0.9 (0.3, 2.0) | 49.0 (-138.6, 91.8) |  | 2.6 (1.6, 3.9) | 3.9 (2.7, 5.4) | 33.6 (-17.3, 63.1) |
| 18C | 0.5  (0.1, 1.3) | 0.5 (0.1, 1.3) | 0.5 (-434.2, 81.5) |  | 0.9  (0.3, 1.9) | 0.4 (0.1, 1.3) | -98.3 (-1125.3, 57.7) |  | 0.5  (0.1, 1.4) | 0.5 (0.1, 1.4) | -1.9 (-660.9, 86.3) |  | 2.1 (1.3, 3.3) | 2.9 (1.9, 4.3) | 28.3 (-36.7, 63.2) |
| 19F | 2.7  (1.7, 4.0) | 4.8 (3.5, 6.6) | 45.0***** (3.9, 69.3) |  | 3.3  (2.1, 4.9) | 4.1 (2.7, 5.8) | 18.6 (-46.6, 55.2) |  | 3.5  (2.2, 5.3) | 3.6 (2.3, 5.4) | 2.5 (-83.0, 48.2) |  | 9.9 (7.9, 12.1) | 14.4 (12.1, 16.9) | 31.5* (8.8, 48.7) |
| 23F | 3.9  (2.7, 5.5) | 4.7 (3.3, 6.4) | 16.6 (-38.1, 50.0) |  | 2.9  (1.8, 4.4) | 2.8 (1.7, 4.3) | -4.4 (-106.7, 47.1) |  | 2.1  (1.1, 3.5) | 4.4 (2.9, 6.3) | 52.7***** (5.6, 77.5) |  | 12.0 (9.9, 14.3) | 14.2 (11.9, 16.7) | 15.4 (-11.1, 35.7) |
| **Any vaccine-related serotype** | 10.3  (8.2, 12.6) | 8.0  (6.2, 10.2) | -27.9  (-80.7, 9.1) |  | 7.9  (6.0, 10.2) | 8.3  (6.3, 10.6) | 4.3  (-41.1, 35.2) |  | 6.1  (4.3, 8.2) | 7.2  (5.3, 9.5) | 15.8  (-32.3, 46.7) |  | 23.0 (20.2, 26.0) | 23.5 (20.7, 26.5) | 2.0 (-20.0, 19.9) |
| **Individual VR serotypes** | | | |  |  |  |  |  |  |  |  |  |  |  |  |
| 6A | 5.2  (3.8, 7.0) | 5.0 (3.6, 6.7) | -4.6 (-66.5, 34.2) |  | 4.0  (2.7, 5.8) | 4.3 (3.0, 6.1) | 7.5 (-60.3, 46.7) |  | 2.4  (1.3, 3.9) | 4.1 (2.7, 5.9) | 41.2 (-15.2, 71.0) |  | 11.6 (9.5, 14.0) | 11.9 (9.8, 14.3) | 2.4 (-30.0, 26.8) |
| 6C | 0.1  (0.0, 0.7) | 0.0  (0.0, 0.5) | I |  | 0.7  (0.2, 1.7) | 0.0  (0.0, 0.5) | I |  | 0.2  (0.0, 0.9) | 0.5  (0.1, 1.4) | 66.0  (-323.1, 99.4) |  | 1.4 (0.7, 2.4) | 0.8 (0.3, 1.7) | -70.6 (-411.5, 38.1) |
| 19A | 2.2  (1.3, 3.4) | 1.1 (0.5, 2.2) | -87.9 (-378.5, 20.8) |  | 1.4  (0.7, 2.6) | 2.0 (1.1, 3.4) | 29.2 (-71.4, 71.9) |  | 1.4  (0.7, 2.7) | 1.1 (0.4, 2.2) | -31.0 (-314.0, 56.6) |  | 5.4 (4.0, 7.1) | 5.1 (3.7, 6.8) | -6.5 (-65.3, 31.3) |
| 23A | 1.9  (1.1, 3.1) | 1.1 (0.5, 2.2) | -65.8 (-329.7, 32.0) |  | 1.0  (0.4, 2.1) | 1.0 (0.4, 2.1) | 0.9 (-231.2, 70.3) |  | 1.3  (0.6, 2.5) | 0.9 (0.3, 2.0) | -35.9 (-375.1, 58.7) |  | 4.1 (2.9, 5.7) | 3.7 (2.5, 5.1) | -12.4 (-88.4, 32.7) |
| **Any other pneumococcal serotype** | 10.8  (8.7, 13.2) | 10.8  (8.8, 13.2) | 0.5  (-36.0, 27.2) |  | 12.8  (10.4, 15.5) | 10.0  (7.9, 12.5) | -27.9  (-77.7, 7.7) |  | 11.8  (9.4, 14.6) | 9.5  (7.4, 12.1) | -23.6  (-76.5, 13.1) |  | 35.2 (32.0, 38.5) | 33.0 (29.9, 36.3) | -6.6 (-26.0, 9.7) |
| ***H. influenzae*** | 5.7  (4.2, 7.6) | 5.6  (4.1, 7.5) | -1.9  (-58.0, 34.3) |  | 4.0  (2.7, 5.8) | 5.5  (3.9, 7.5) | 27.0  (-22.2, 56.8) |  | 4.6  (3.1, 6.6) | 5.2  (3.6, 7.2) | 10.4  (-52.1, 47.5) |  | 19.7 (17.1, 22.5) | 21.5 (18.7, 24.4) | 8.3 (-13.7, 26.1) |
| Non-typeable  *H. influenzae* | 5.7  (4.2, 7.6) | 5.5  (4.0, 7.3) | -4.3  (-62.2, 32.9) |  | 4.0  (2.7, 5.8) | 5.5  (3.9, 7.5) | 27.0  (-22.2, 56.8) |  | 4.6  (3.1, 6.6) | 5.0  (3.4, 7.0) | 7.6  (-57.6, 46.1) |  | 19.4 (16.8, 22.3) | 21.0 (18.3, 23.9) | 7.4 (-15.1, 25.5) |
| ***Staphylococcus aureus*** | 8.1  (6.3, 10.2) | 6.0  (4.4, 7.9) | -35.7  (-102.1, 8.4) |  | 7.3  (5.5, 9.5) | 6.8  (5.0, 9.0) | -7.6  (-63.4, 29.1) |  | 6.5  (4.7, 8.8) | 6.3  (4.5, 8.4) | -4.5  (-65.7, 34.1) |  | 22.6 (19.8, 25.6) | 24.9 (22.0, 27.9) | 9.2 (-10.9, 25.7) |
| ***Streptococcus pyogenes*** | 0.0  (0.0, 0.5) | 0.1  (0.0, 0.7) | 100 (-3785.2 , 100) |  | 0.4  (0.1, 1.3) | 0.0  (0.0, 0.5) | I |  | 0.0  (0.0, 0.6) | 0.0  (0.0, 0.6) | U |  | 0.6 (0.2, 1.4) | 0.1 (0.0, 0.7) | -396.5 (-23382.6, 44.4) |
| ***Moraxella catarrhalis*** | 0.1  (0.0, 0.7) | 0.0  (0.0, 0.5) | I |  | 0.1  (0.0, 0.8) | 0.3  (0.0, 1.0) | 50.4  (-852.2, 99.2) |  | 0.5  (0.1, 1.4) | 0.8  (0.3, 1.8) | 38.9  (-214.3, 90.5) |  | 0.9 (0.4, 1.8) | 1.2 (0.6, 2.2) | 20.6 (-123.5, 72.8) |

******P*-value (two-sided conditional exact test) <0.05 without any correction for multiplicity.

N indicates number of children with swabs cultured at the specified time point or (for overall) number of swabs cultured after at least one visit.

^a^N = 789 for *S. aureus*, *S. pyogenes*, *M. catarrhalis*.

^b^N = 785 for *H. influenzae* and non-typeable *H. influenzae;* N = 786 for *S. aureus*, *S. pyogenes*, *M. catarrhalis.*

^c^N = 628 for *H. influenzae*, non-typeable *H. influenzae,* *S. aureus*, *S. pyogenes*, *M. catarrhalis.*

^d^N = 640 for *H. influenzae*, non-typeable *H. influenzae,* *S. aureus*, *S. pyogenes*, *M. catarrhalis.*^e^N = 854 for *H. influenzae*, non-typeable *H. influenzae,* *S. aureus*, *S. pyogenes*, *M. catarrhalis.*

U, undefined; I, negative infinity; 95% CI, 95% confidence interval.
