# Supplementary material for: Efficacy of 10-valent pneumococcal non-typeable Haemophilus influenzae protein D conjugate vaccine against acute otitis media and nasopharyngeal carriage in Panamanian children – A randomized controlled trial
Source: Hum Vaccin Immunother. 2017 Feb 25;13(6):1213–28. doi: 10.1080/21645515.2017.1287640 (PMC5489287; doi:10.1080/21645515.2017.1287640)
Supplement: Supplemental_Material.zip [file khvi-13-06-1287640-s001.zip › Supplemental digital content 4.docx]

**Supplemental digital content 4.** Acquisition of nasopharyngeal carriage of *S. pneumoniae*, *H. influenzae* or other bacterial pathogens, and vaccine efficacy against acquisition across all time points (intent-to-treat cohort).

|  | **PHiD-CV group  (N = 812)** | | **Control group  (N = 809)** | | **Vaccine efficacy, % (95% CI)** |
| --- | --- | --- | --- | --- | --- |
|  | **N** | **% (95% CI)** | **n** | **% (95% CI)** |  |
| Any pneumococcal serotype | 440 | 54.2 (50.7, 57.7) | 474 | 58.6 (55.1, 62.0) | 7.5 (-5.5, 19.0) |
| Any PHiD-CV serotype | 197 | 24.3 (21.3, 27.4) | 277 | 34.2 (31.0, 37.6) | 29.1 (14.6, 41.3) |
| Serotype 6B | 53 | 6.5 (4.9, 8.5) | 82 | 10.1 (8.1, 12.4) | 35.6 (7.9, 55.3) |
| Serotype 14 | 13 | 1.6 (0.9, 2.7) | 26 | 3.2 (2.1, 4.7) | 50.2 (-0.5, 76.5) |
| Serotype 18C | 14 | 1.7 (0.9, 2.9) | 17 | 2.1 (1.2, 3.3) | 18.0 (-76.9, 62.6) |
| Serotype 19F | 57 | 7.0 (5.4, 9.0) | 88 | 10.9 (8.8, 13.2) | 35.5 (8.9, 54.6) |
| Serotype 23F | 73 | 9.0 (7.1, 11.2) | 86 | 10.6 (8.6, 13.0) | 15.4 (-16.9, 39.0) |
| Any vaccine-related serotype | 146 | 18.0 (15.4, 20.8) | 147 | 18.2 (15.6, 21.0) | 1.0 (-25.3, 21.8) |
| Serotype 6A | 69 | 8.5 (6.7, 10.6) | 69 | 8.5 (6.7, 10.7) | 0.4 (-41.2, 29.7) |
| Serotype 6C | 7 | 0.9 (0.3, 1.8) | 5 | 0.6 (0.2, 1.4) | -39.5 (-457.3, 61.9) |
| Serotype 19A | 34 | 4.2 (2.9, 5.8) | 33 | 4.1 (2.8, 5.7) | -2.6 (-71.0, 38.3) |
| Any other pneumococcal serotype | 226 | 27.8 (24.8, 31.1) | 224 | 27.7 (24.6, 30.9) | -0.5 (-21.5, 16.8) |
| *H. influenzae* | 131 | 16.1 (13.7, 18.8) | 149 | 18.4 (15.8, 21.3) | 12.4 (-11.5, 31.3) |
| Non-typeable *H. influenzae* | 131 | 16.1 (13.7, 18.8) | 147 | 18.2 (15.6, 21.0) | 11.2 (-13.1, 30.4) |
| *Staphylococcus aureus* | 171 | 21.1 (18.3, 24.0) | 184 | 22.7 (19.9, 25.8) | 7.4 (-14.6, 25.3) |
| *Streptococcus pyogenes* | 4 | 0.5 (0.1, 1.3) | 1 | 0.1 (0.0, 0.7) | -298.5 (-19526.2, 60.6) |
| *Moraxella catarrhalis* | 6 | 0.7 (0.3, 1.6) | 9 | 1.1 (0.5, 2.1) | 33.6 (-108.9, 80.5) |

N, number of children with swabs cultured after at least one visit; n, number of children with new bacterial acquisition after at least one visit, 95% CI, 95% confidence interval.
