# Supplementary material for: Efficacy of 10-valent pneumococcal non-typeable Haemophilus influenzae protein D conjugate vaccine against acute otitis media and nasopharyngeal carriage in Panamanian children – A randomized controlled trial
Source: Hum Vaccin Immunother. 2017 Feb 25;13(6):1213–28. doi: 10.1080/21645515.2017.1287640 (PMC5489287; doi:10.1080/21645515.2017.1287640)
Supplement: Supplemental_Material.zip [file khvi-13-06-1287640-s001.zip › Supplemental digital content 5.docx]

**Supplemental digital content 5. Methodology of AOM surveillance**

Initially, AOM cases were captured when parents sought medical attention for children with symptoms of AOM. However, because of a lower than expected AOM rate, the surveillance was enhanced approximately 2 years into the study (from July 2009) through regular telephone calls or home visits by study personnel, who advised parents to visit the clinic if their child had symptoms suggestive of AOM. During the entire study period, once a case was captured and if AOM was suspected by the physician, the child was referred for confirmation to one of the ENT specialists involved in the trial.
