# Supplementary material for: Efficacy of 10-valent pneumococcal non-typeable Haemophilus influenzae protein D conjugate vaccine against acute otitis media and nasopharyngeal carriage in Panamanian children – A randomized controlled trial
Source: Hum Vaccin Immunother. 2017 Feb 25;13(6):1213–28. doi: 10.1080/21645515.2017.1287640 (PMC5489287; doi:10.1080/21645515.2017.1287640)
Supplement: Supplemental_Material.zip [file khvi-13-06-1287640-s001.zip › Supplemental digital content 6.docx]

**Supplemental digital content 6. Ethical considerations and informed consent**

The trial was designed in accordance with ICH Good Clinical Practice (GCP) guidelines and the 1996 Declaration of Helsinki. During the course of the study, when issues relating to compliance with GCP guidelines or the protocol were identified, they were investigated and corrective actions were implemented consistent with GCP guidelines and in order to achieve compliance with the protocol.

An Independent Data Monitoring Committee (IDMC) supervised study progress and safety of the children by reviewing serious adverse events, assessing potential treatment harm, and making recommendations to GSK Vaccines regarding safety measures, study design, and reporting or analysis plans. The committee included seven external experts in infectious diseases or statistics from Latin America, the USA, Europe, and Africa, and met at least every 6 months, depending on study stage.

In Panama, there were two separate issues involving the informed consent process. In both cases, the situations were discussed with the IDMC, which agreed with the corrective actions described below. Ethics committees and competent authorities were consulted accordingly.

1. During the early phases of study enrolment, there was a lack of clarity in Panamanian law regarding the ability of minor parents to provide informed consent to let their child participate in a clinical study. Prompted by the findings in Colombia,^1^ a local law firm and the Institutional Review Board were consulted. The sponsor was advised and made all possible efforts to follow the process to obtain consent from the grandparents and to re-consent parents when they reached legal age. Therefore, the data of these children were used for the interim analysis on the primary objective of this study. However, during quality check and re-monitoring activities initiated later in 2011, it was found that re-consent had, in fact, not been obtained from some minor parents when they reached the legal age and in some cases, grandparents had not been asked to confirm consent. The IRB was informed and recommended additional attempts to obtain re-consent from minor parents when they reached the legal age and, in the event re-consent could not be obtained, the IRB agreed to allow use of the data. In summary, parents of 150 children re-consented, parents of 60 children could not be re-contacted or did not agree to re-consent and, for 53 children, the parents’ age could not be confirmed. In addition, original informed consent forms signed by parents of 31 children in Panama were lost during re-monitoring activities. In line with guidance from the European Medicines Agency, a descriptive sensitivity analysis was conducted on the primary objective in which children with informed consent issues were excluded to confirm the validity of the interim analysis. Also, children with informed consent issues were excluded from the end of study analyses.
2. In addition, it was discovered that an incorrect version of the informed consent form for the immunogenicity subset was used in Panama. Specifically, parents were asked to sign a version of the form that included all information on the study procedures related to efficacy endpoints but inadvertently did not specify the collection of the blood samples required for the immunogenicity analyses and completion of diary cards required for the reactogenicity analyses. However, the collection of blood samples and completion of diary cards were explained verbally to parents during the informed consent process and the children followed study procedures relevant for the immunogenicity subset during the study. When the error was detected, in agreement with the Independent Ethics Committee, parents were re-contacted to confirm their agreement to the use of the immunogenicity data for their child. For 262 children, parents or guardians could not be contacted to provide consent or did not agree to the use of immunogenicity data. In addition, two children were excluded because the original informed consent forms were lost during the re-monitoring activities. Therefore, 264 children were excluded from the intent-to-treat cohort for immunogenicity.

For suspected acute otitis media (AOM) cases, the Ear, Nose and Throat (ENT) specialist was to decide if a middle ear fluid (MEF) sample could be collected. Prior to collection of the MEF sample, the ENT specialist was to obtain a specific informed consent for tympanocentesis from the parents or guardians of the child. However, for 6 MEF samples collected from 6 children, either the informed consent form was lost (3 samples) or no informed consent form was obtained (3 samples). One of these MEF samples was collected from spontaneous drainage according to hospital routine practice; the Independent Ethics Committee was informed and approved the use of data related to this MEF sample for analysis. Data related to the other 5 MEF samples were not used for analysis of bacteriologically confirmed AOM (B-AOM).
