# Supplementary material for: Efficacy of 10-valent pneumococcal non-typeable Haemophilus influenzae protein D conjugate vaccine against acute otitis media and nasopharyngeal carriage in Panamanian children – A randomized controlled trial
Source: Hum Vaccin Immunother. 2017 Feb 25;13(6):1213–28. doi: 10.1080/21645515.2017.1287640 (PMC5489287; doi:10.1080/21645515.2017.1287640)
Supplement: Supplemental_Material.zip [file khvi-13-06-1287640-s001.zip › Supplemental digital content 7.docx]

**Supplemental digital content 7. Microbiological procedures**

MEF and spontaneous otorrhea samples, as well as nasopharyngeal swabs were inoculated into transport medium (Amies agar gel without charcoal) and sent within 12 hours to the local laboratory for bacteriological culture. Samples were plated and incubated on blood and chocolate agar. If *S. pneumoniae* or *H. influenzae* was detected, the isolate was sent to the central laboratory (Eurofins Medinet, Inc., Herndon, VA, USA) for confirmation of identity, serotyping and antibiotic sensitivity testing. Therefore, *S. pneumoniae* or *H. influenzae* colonies were inoculated into a cryoconservation medium and kept at ‑70°C. At least 3–5 days before shipment to the central laboratory, these bacterial samples were sub-cultured and incubated again on blood or chocolate agar as appropriate. Isolated colonies were inoculated into transport medium (Amies with charcoal for dispatch of *S. pneumoniae* and without charcoal for dispatch of *H. influenzae*) and sent to the central laboratory. Other otopathogens and their antibiotic sensitivity were only confirmed at the local microbiology lab.

*H. influenzae*, *S. pneumoniae,* *S. aureus*, *S. pyogenes* and *M. catarrhalis* were identified using standard bacteriological procedures.^2^ The absence of slide agglutination in the presence of antisera to types a–f (*H. influenzae* Agglutinating Sera MUREX ZM 20-25) was used to identify non-encapsulated (non-typeable) *H. influenzae* strains. *S. pneumoniae* serotypes were identified using latex agglutination, and by Quellung reaction using pooled antisera and selected factor antisera (Statens Serum Institute, Copenhagen, Denmark). Discrimination between pneumococcal serotypes 6A and 6C was performed by Quellung reaction using antiserum specific for 6C.

Standard laboratory methods do not reliably distinguish strains of *H. influenzae* from strains of *H. haemolyticus,* a respiratory tract commensal.^3^ In the current study, duplex lgtC/P6 real-time polymerase chain reaction (PCR) test was conducted at GSK Vaccines’ laboratory in Rixensart, Belgium to distinguish *H. influenzae* from *H. haemolyticus* strains in *H. influenzae* isolates obtained from nasopharyngeal swabs. The lgtC gene is ubiquitous in *H. influenzae* strains and is 54 times less prevalent in *H. haemolyticus* than in *H. influenzae*.^4^ Protein P6, which is the internal control for PCR, is shared by both *H. influenzae* and *H. haemolyticus* (differ by 4 of 53 amino acids).^3^ The P6 primers and the probe set were designed from a DNA region conserved in both species. Bacterial isolates identified *as H. influenzae* were thawed, heated at 95°C for 5 min and centrifuged at 8,000 rpm for 10 min; 5 µL of the supernatant was used for the PCR reaction as template DNA. Real-time PCR was performed in a 25 µL volume containing TaqMan 2X Universal PCR Master Mix (Applied Biosystems). Reactions were performed on an ABI PRISM® 7900HT Sequence Detection System (Applied Biosystems) using universal cycling parameters. The sequences and final concentrations of oligonucleotides are provided in Supplemental Digital Content 8.
