# Supplementary material for: Efficacy of 10-valent pneumococcal non-typeable Haemophilus influenzae protein D conjugate vaccine against acute otitis media and nasopharyngeal carriage in Panamanian children – A randomized controlled trial
Source: Hum Vaccin Immunother. 2017 Feb 25;13(6):1213–28. doi: 10.1080/21645515.2017.1287640 (PMC5489287; doi:10.1080/21645515.2017.1287640)
Supplement: Supplemental_Material.zip [file khvi-13-06-1287640-s001.zip › Supplemental digital content 8.docx]

**Supplemental digital content 8. Statistical analysis**

The first secondary confirmatory objective was to demonstrate, in the per-protocol cohort, positive PHiD-CV vaccine efficacy (VE against first C-AOM episodes reported from 2 weeks after dose 3 (non-adjusted one-sided test; nominal type I error of 2.5%). The number of first C-AOM episodes required for this objective was calculated according to various true VE assumptions. If VE was 20%, 662 and 880 first C-AOM episodes were required to demonstrate positive VE with 80% and 90% power, respectively (non-adjusted one-sided test; nominal type I error of 2.5%). If VE was 15%, 1226 and 1637 first C-AOM episodes were required for 80% and 90% power, respectively.

PHiD-CV VE against AOM was assessed in the per-protocol cohort and the intent-to-treat cohort for AOM. The per-protocol cohort for AOM comprised participants who had complied with study procedures and did not meet elimination criteria. In the per-protocol analysis, follow-up starting 2 weeks after the third primary dose was censored at whatever came first: end of the study, time of last contact (in case of withdrawal), time of unblinding, time of booster vaccination (if the booster dose was not given correctly), or 18 months of age (if the booster dose was not given by this age). The intent-to-treat follow-up started at the time of first vaccination without censoring procedure.

The percentage of children who experienced at least one AOM episode and the incidence of all AOM episodes, including subsequent episodes in the same child, were calculated. VE was calculated as (1 – hazard ratio) x 100 using a Cox (first AOM episodes) or generalized Cox (all AOM episodes) regression model.

**References**

1. Tregnaghi MW, Saez-Llorens X, Lopez P, Abate H, Smith E, Posleman A, Calvo A, Wong D, Cortes-Barbosa C, Ceballos A, et al. Efficacy of pneumococcal nontypable Haemophilus influenzae protein D conjugate vaccine (PHiD-CV) in young Latin American children: A double-blind randomized controlled trial. PLoS Med 2014; 11: e1001657.

2. Murray PR, Baron EJ, Jorgensen JH. Manual of clinical microbiology. (9th ed.) Washington, D.C.: American Society for Microbiology. 2007.

3. Murphy TF, Brauer AL, Sethi S, Kilian M, Cai X, Lesse AJ. Haemophilus haemolyticus: a human respiratory tract commensal to be distinguished from Haemophilus influenzae. J Infect Dis 2007; 195: 81-89.

4. McCrea KW, Xie J, LaCross N, Patel M, Mukundan D, Murphy TF, Marrs CF, Gilsdorf JR. Relationships of nontypeable Haemophilus influenzae strains to hemolytic and nonhemolytic Haemophilus haemolyticus strains. J Clin Microbiol 2008; 46: 406-416.
